# Supplementary material for: Protocol for a cluster randomised waitlist-controlled trial of a goal-based behaviour change intervention for employees in workplaces enrolled in health and wellbeing initiatives
Source: PLoS One. 2023 Sep 28;18(9):e0282848. doi: 10.1371/journal.pone.0282848 (PMC10538707; doi:10.1371/journal.pone.0282848)
Supplement: S1 File — (DOCX) [file pone.0282848.s001.docx]

# S1 – Systematic literature search PRISMA flow diagram, databases, and search string

Figure 1: PRISMA flow diagram of studies included in systematic review of workplace trials using mental contrasting (without or with implementation intentions). Note that ‘studies’ are included within ‘reports’ in accordance with PRISMA guidance.[17]
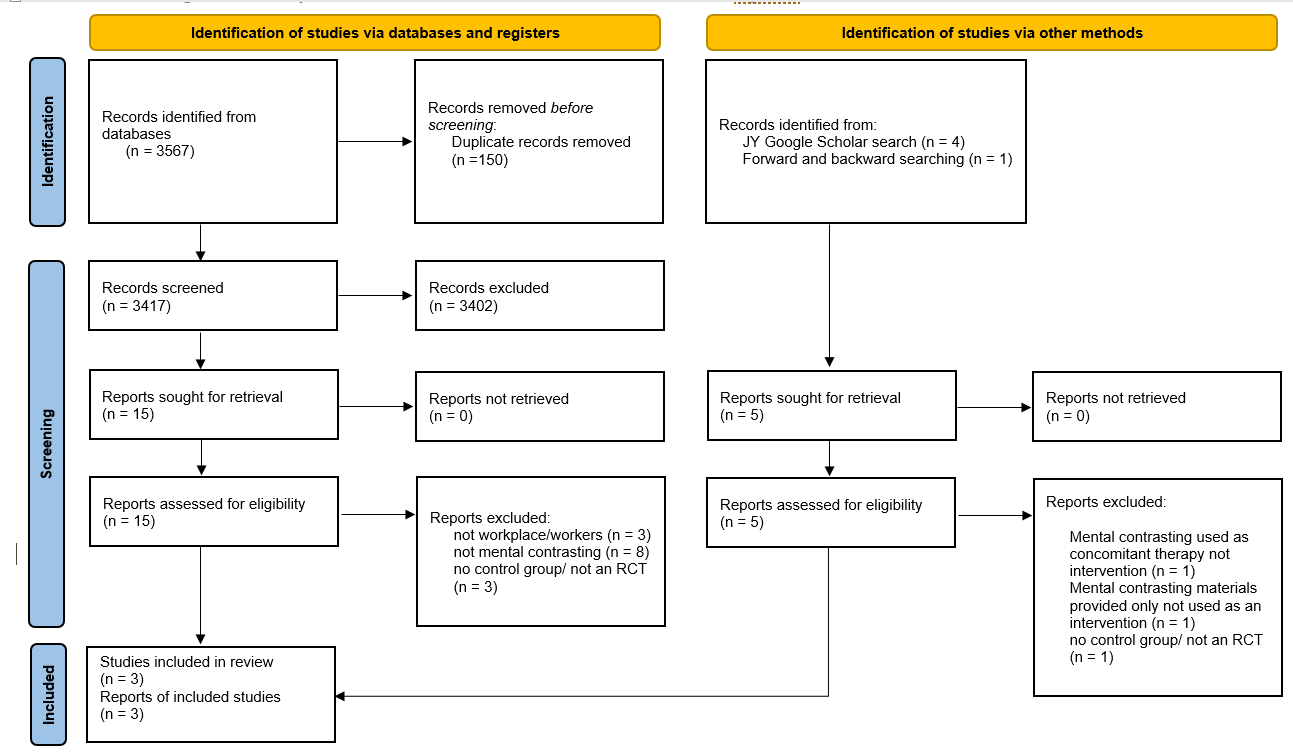


Databases searched were:

 Business Source Premier. EBSCO (Firm),

 Emerald Insight. Emerald Publishing Corporation.

 MEDLINE. Wolters Kluwer (Firm).

 SCOPUS. Elsevier (Firm).

 PsycINFO. Wolters Kluwer (Firm).

 Web of Science (All Databases). Clarivate Analytics.

 ABI/INFORM Global. ProQuest (firm).

 CINAHL Plus. EBSCO (Firm).


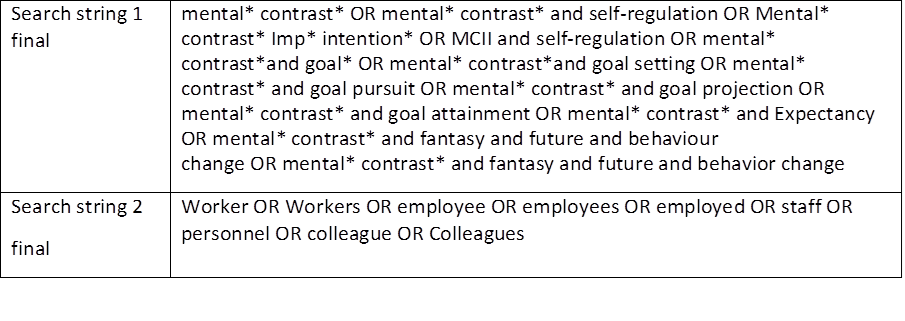


Conducted on 08/12/21
