# Supplementary material for: Protocol for a cluster randomised waitlist-controlled trial of a goal-based behaviour change intervention for employees in workplaces enrolled in health and wellbeing initiatives
Source: PLoS One. 2023 Sep 28;18(9):e0282848. doi: 10.1371/journal.pone.0282848 (PMC10538707; doi:10.1371/journal.pone.0282848)
Supplement: S3 File — a. Session 1 ‐ Part 1 –all. b. Session 1 ‐ Part 2 ‐ intervention only. c. Session 1 ‐ Part 2 ‐ control only. d. Session 2 ‐ intervention only. e. Session 2 ‐ control only. f. Handouts. (ZIP) [file pone.0282848.s003.zip › S3a. Session 1 - Part 1 - all.pdf]

Part 1 – all

Welcome

-Introduce self - name, job title [redacted] working with [redacted]

-Explain purpose – this session is about going from 'want' to 'act'.

Many people know what they want to do to be healthier and happier – but can find it hard. They might take on too much or give up too soon.

**Interaction

Have a think, how many of you have wanted to do something to improve your health and wellbeing but it has not happened in the end? A show of hands?

In this session we will learn a new technique from the science of goal setting for your health and wellbeing.

[redacted]

Survey

No right or wrong answers

Fill out on your own

Your individual responses are not looked at by others in your workplace

We want to be sure these sessions are working so before we start, I would like to ask you to please fill out a survey about your perceptions of health and wellbeing. I will give you the link/questionnaire in a moment.

In the survey, there are no right or wrong answers.

It is something for you to fill out on your own, you do not need to talk to anyone else about your answers.

It is completely confidential and will not be looked at by anyone else in your workplace, only by researchers like me working on the research.

I am going to put a link to the survey in the chat now / give out paper copies.

**There is also an information sheet to answer any questions you have about the survey. I can answer any questions you have, too.

[redacted]

**Hand out W sheet as people complete surve [redacted]

**Once done, check that everyone was able to complete the survey – if not, they can still take part in the session, but we will need to know and record that they opted out

[redacted]

Part of the reason that I am here today is because your organisation is signed up to something called T[redacted]

Has anyone heard o [redacted] [ask for response and clarify using details below as needed]

[redacted] is a programme which provides guidance for organisations on creating a workplace that promotes employee wellbeing.

Organisations like yours can receive support and guidance on how to invest in employees' health and wellbeing – such as how to provide information, activities, and services.

Create space to think

The first part of this session is about slowing down and taking time to create the space to really think. Make sure you are feeling comfortable – as much as you can be. You might like to put away anything that might be distracting, like phones or laptops. Take a moment to relax and slow down as we work through the session. You might like to take a breath in, and then a breath out.

Even though this is a group session, it is about what you as an individual can do – it is for you. Do not think too much about the person next to you.

This might be different to other exercises you have been involved in because it involves thoughts and images rather - rational or effortful thinking.

It involves going slow, and making room within yourself for imagining.

The idea is to start when you feel calm, slow and comfortable. This is your time now. Clear your mind and create space to imagine.

***Make sure everyone as W sheet (hand out W sheet as people complete survey – see prior slide about the survey) -

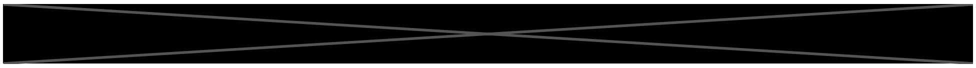

Wish

The first bit of this session is about a Wish.

There are many different things that people might wish to do for their health and wellbeing.

As people go about their daily activities, from getting ready in the morning to going to sleep at night, various things might arise, and they might different have wishes and concerns about what they would like to change.

Think about what health and wellbeing means for you – not what others want you to do, but what you truly wish for in your life.

The wish should be about your health and wellbeing, but this can be an any area, it does not have to be about your work and profession – it could be your relationships or what you do outside of work, too. It could be any life domain.

Think about one wish or concern that you would like to address or achieve in the next four weeks.

What your most important personal wish – one that you would like to see yourself accomplish by the next four weeks?

Fulfilling your wish or solving your concern should be challenging for you, but you should also feel it is possible for you to achieve your wish in the next four weeks.

If you have several concerns or wishes, select the one that is most important to you.

Find your wish and keep it in the front of your mind.

Wish

Take a moment and think about the next four weeks. Consider, what is one wish or concern you have about your health and wellbeing? It does not have to be about work or your profession. Choose something that feels challenging to you but also that you feel is achievable in the next four weeks.

Note your wish using no more than a few sentences:

____

Take a moment to read about a wish and to write yours down.

[Count to 10 – wait 10 seconds before proceeding]

Wish - check

Is this wish something that is truly important to you?

Now, check your wish. Is it something that is truly important to you? If not, please take the time to revise your wish, thinking of something important to you. It should come from within yourself, rather than what other people expect you to do. It does not have to be about work.

[Count to 10 – wait 10 seconds before proceeding]

Wish - check

Do you believe it is possible for you to achieve it in four weeks?

Now check, again - is it something do you really think you can possible achieve in four weeks? If your wish could be more achievable, try to revise it into something achievable.

[Count to 10 – wait 10 seconds before proceeding]

Wish - check

Is it challenging for you – not too easy and not too hard?

Now check, is it something that is challenging for you? It should not be something that feels too easy, or something that you think would be too hard and impossible for you to do. If not, perhaps revise it a bit.

[Count to 10 – wait 10 seconds before proceeding]

Wish - check

Did you summarize it using no more than a few sentences?

Finally, did you summarise your wish using no more than a few sentences? If not, take a moment to revise it now.

[Count to 10 – wait 10 seconds before proceeding]

Take some time to reflect on your wish
(around one minute)

Now, take some time to reflect on your wish. Really keep it at the front and centre of your thoughts.

Open envelope/email

Control group – next slide

Intervention group ONLY – Part 2
