# Supplementary material for: Protocol for a cluster randomised waitlist-controlled trial of a goal-based behaviour change intervention for employees in workplaces enrolled in health and wellbeing initiatives
Source: PLoS One. 2023 Sep 28;18(9):e0282848. doi: 10.1371/journal.pone.0282848 (PMC10538707; doi:10.1371/journal.pone.0282848)
Supplement: S3 File — a. Session 1 ‐ Part 1 –all. b. Session 1 ‐ Part 2 ‐ intervention only. c. Session 1 ‐ Part 2 ‐ control only. d. Session 2 ‐ intervention only. e. Session 2 ‐ control only. f. Handouts. (ZIP) [file pone.0282848.s003.zip › S3b. Session 1 - Part 2 - intervention only.pdf]

Part 2

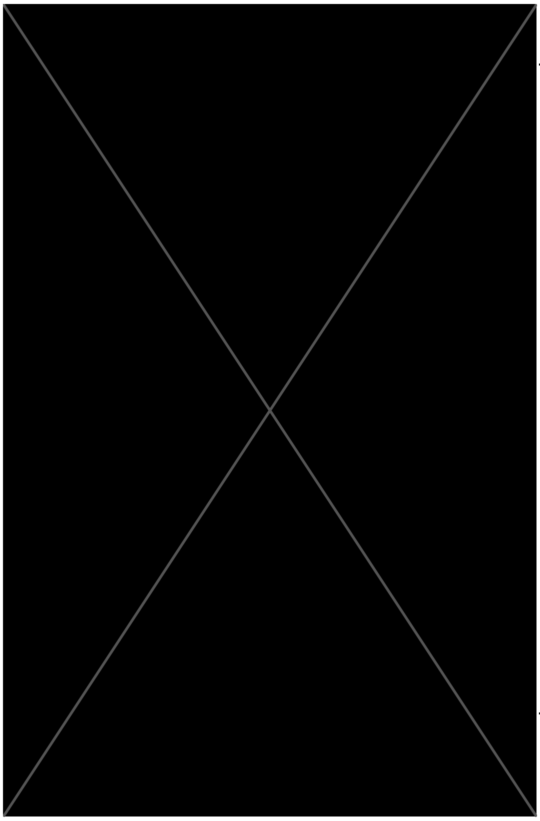

Lets move onto the next stages

Outcome

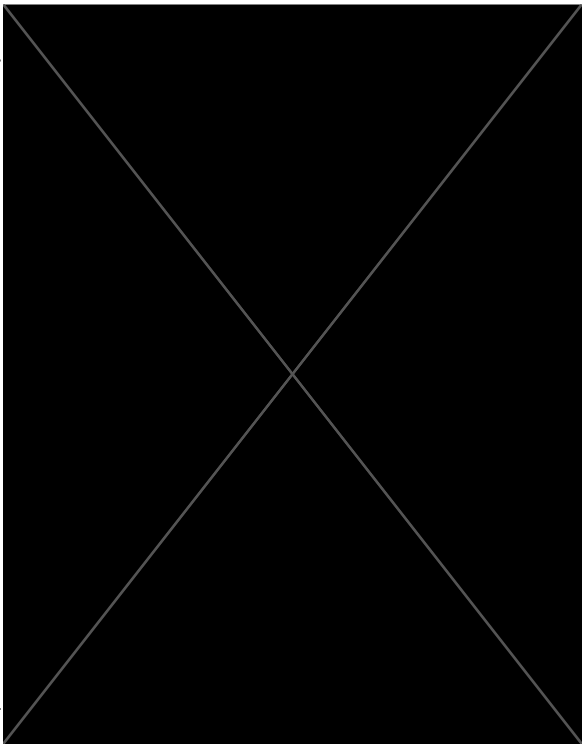

Now, what would be the best thing – the best outcome - you associate with fulfilling your wish?

How would you feel if your wish were to happen?

Take a moment and imagine this outcome vividly and in detail, and write down your thoughts and ideas. Again, using no more than a few sentences.

Outcome

What would be the best thing – the best outcome – of fulfilling your wish? How would you feel if you were to fulfil your wish?

Note your best Outcome using no more than a few sentences:

____

After you are done writing, really focus on imagining this outcome.

Imagine this best outcome vividly and in detail

[Count to 20]

Obstacle

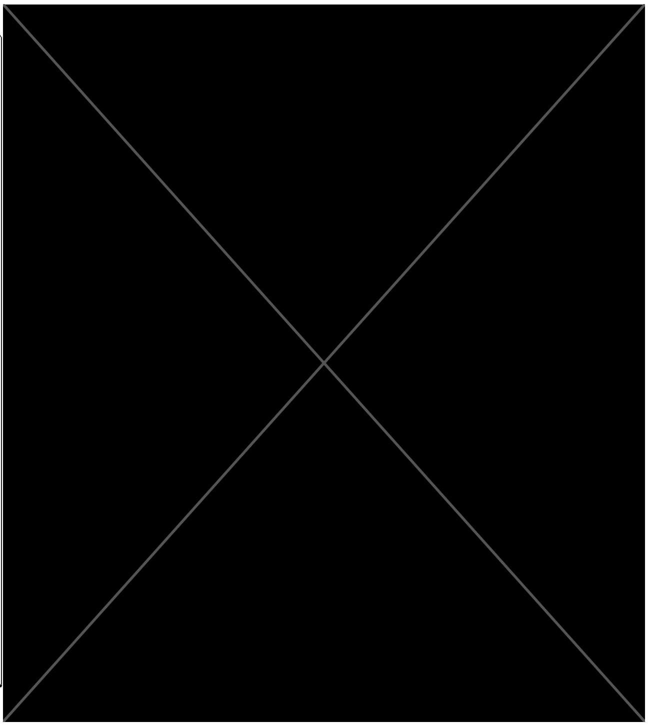

Now, lets continue.

Obstacle

Which is the main obstacle that prevents you from fulfilling your wish?

What is it *within you* that stops or hinders you from fulfilling your wish?

What is your inner obstacle – something that you need to overcome for your wish to happen?

It might be a feeling, something you do or a habit, or a thought think or a belief you have.

Please think twice, is this the real, inner personal obstacle? Dig deeper – what is it in you that is holding you back?

What is your one main obstacle?

Find it. Again, please write down your thoughts, using no more than a few sentences.

Obstacle

What is it ***within you*** that stops or hinders you from fulfilling your wish? What is your inner obstacle – something that you need to overcome for your wish to happen? It might be a feeling, something you do or a habit, or a thought think or a belief you have. Please think twice, is this the real, inner personal obstacle? Dig deeper – what is it in you that is holding you back?

Note your main inner Obstacle, again using no more than a few sentences: ____

After you are done writing, really focus on imagining your main inner personal obstacle occurirng.

Imagine your main inner obstacle vividly and in detail

[Count to 20]

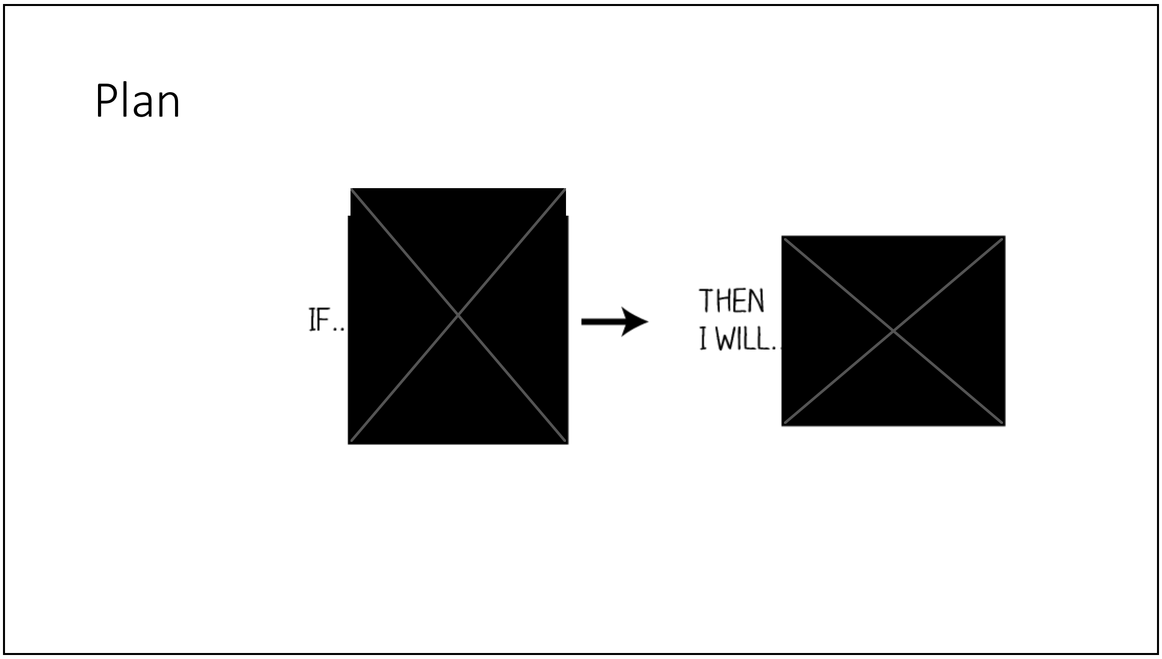

Now, lets continue.

Plan

Now - what can you do to overcome your obstacle?

Think about an effective action you can take, or something you can think, to overcome the obstacle.

What can you do in order to overcome the obstacle? Something that is in your control. How can you respond?

One effective action you can take or something you can think.

Plan

What can you do to overcome your obstacle? Think about an effective action you can take, or something you can think, to overcome the obstacle.

Note your action or thought using no more than a few sentences:

____

Specify it, and again, write it down.

Make an If-Then plan:

If… (obstacle) then I will … (action/thought).

Fill in the blanks yourself:

If… __________________, then I will ___________

Repeat your if-then plan and really imagine it.

Now, we are going to make an if-then plan.

First, write down the obstacle you found earlier after the word 'If'.

'If, obstacle'.

Next, write down the action or thought you found earlier to overcome your obstacle. Put it after the words ‘then I will’.

So, reading it over, this is - if (your obstacle), then I will (your action or thought to overcome the obstacle).

Read your if-then a few times – and imagine it:

If (now you imagine the obstacle), then I will (and now you imagine the behaviour to overcome the obstacle).

Really take the time to imagine it.

Again, if (obstacle – perhaps even thinking about when and where it happens), then I will (specify behaviour to overcome the obstacle).

Take a few moments now to practice this in your mind.

[Allow 20 seconds]

That’s it - WOOP

- Wish
- Outcome
- Obstacle
- Plan

That’s it – WOOP – Wish, Outcome, Obstacle, Plan – a four step way to motivate yourself to make changes that you want to for your health and wellbeing.

You can use WOOP as much or as little ask you like – for small wishes and big wishes, short term wishes, or long term wishes. You could use WOOP when you feel like you have too much on your plate or when there is something you would really like to do for yourself.

Some people say they use WOOP everyday and that it helps them in their everyday life and plans for their future. Do not worry if every wish you have does not happen, though. People who use WOOP say they have seen improvements in their work, family life, and relationships.

If you use WOOP often, and practice it, you will get better at it. So really get to know it - it can help you to find out what it is you really want and what is it that keep you from going for what you really want. You can go from 'wanting' - and knowing more about your 'wanting' - to doing.

Thank you for taking part.

Your perception of usefulness of WOOP

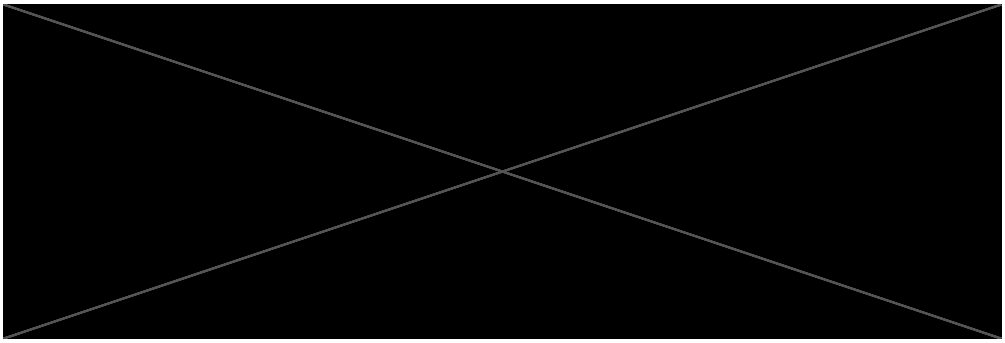

**Interaction

So now that you have done a WOOP, please take a moment to think – what is your perception of the usefulness of WOOP? Low, medium, or high usefulness?

On a scale from 1 to 10, 1 being low, 5-6 medium, and 10 is high, what is your perception? Show me with your hands/fingers

If someone scored <5, ask them what can be done to increase how useful they find it

Thank you for your suggestions, record practical suggestions or apologise that they will not be able to be included.

Suggested commentary, reflections and questions - [redacted] or Group Discussion

- Why is might making a wish or considering a concern in this way be important?
- Why is might making a wish or considering obstacles, outcomes, and plans in this way be important?
- What sort of benefit could WOOP have?
- What was the lead's WOOP (if they are willing to share?)

**Interaction

Thank you

When is your next session?

Thank you all for attending today's session.

As a reminder your next session is X time X date.

Do you have any questions for me?

OOP handout to share:
[redacted]
