# Supplementary material for: Protocol for a cluster randomised waitlist-controlled trial of a goal-based behaviour change intervention for employees in workplaces enrolled in health and wellbeing initiatives
Source: PLoS One. 2023 Sep 28;18(9):e0282848. doi: 10.1371/journal.pone.0282848 (PMC10538707; doi:10.1371/journal.pone.0282848)
Supplement: S3 File — a. Session 1 ‐ Part 1 –all. b. Session 1 ‐ Part 2 ‐ intervention only. c. Session 1 ‐ Part 2 ‐ control only. d. Session 2 ‐ intervention only. e. Session 2 ‐ control only. f. Handouts. (ZIP) [file pone.0282848.s003.zip › S3c. Session 1 - Part 2 - control only.pdf]

Reflections and suggested questions [redacted]
[redacted] or Group Discussion

- How is creating a wish or goal in this way different to other approaches?
- What sort of benefit could making wishes and goals in this way have?
- What did the lead wish for (if they are willing to share?)

**Interaction

That’s it for today

- When is the next session?
- Next time we will come back to your wish
- For the next few weeks, try not to forget about your wish – in the next session, we will work on new ways to make it more likely to happen
- Be aware that there are ways you can access health and wellbeing support, if needed

A reminder of W – [redacted]

Thank you
