# Supplementary material for: Protocol for a cluster randomised waitlist-controlled trial of a goal-based behaviour change intervention for employees in workplaces enrolled in health and wellbeing initiatives
Source: PLoS One. 2023 Sep 28;18(9):e0282848. doi: 10.1371/journal.pone.0282848 (PMC10538707; doi:10.1371/journal.pone.0282848)
Supplement: S3 File — a. Session 1 ‐ Part 1 –all. b. Session 1 ‐ Part 2 ‐ intervention only. c. Session 1 ‐ Part 2 ‐ control only. d. Session 2 ‐ intervention only. e. Session 2 ‐ control only. f. Handouts. (ZIP) [file pone.0282848.s003.zip › S3d. Session 2 - intervention only.pdf]

Welcome back

-Introduce self - name, [redacted] note who they saw last time and that you are here now for Session 2

-Explain purpose – this session is about discussing WOOP and understanding why it worked for you or not

-Give survey **to add link**

Discussion

- What was your general impression of WOOP?
- What do you think is the difference between WOOP and what else is offered at work for health and wellbeing?
- What was the purpose of WOOP and what was required of you when taking part?
- How could WOOP affect your health and wellbeing?

**Interaction

I would like us to take some time to talk about wish, outcome, obstacle and plan, also called ‘woop’ or ‘mental contrasting with implementation intentions’ [if needed, recall this is something that we practiced several weeks ago, which was about how to think about the future and any obstacles related to what you wish to do for your health and wellbeing]

Part 1 – Normalisation process theory items on mechanisms

Part A - coherence
What do you think is the difference between woop and what is offered at work?
What was the purpose of woop and what was required of you when taking part?
How would/did woop affect/influence your health and wellbeing?

Discussion

- What do you think could help sustain or improve WOOP?
- Would you incorporate it into your work life? *Why and why not?*
- Would you incorporate it into your life outside of work? *Why and why not?*

**Interaction

Part B - cognitive participation
What will help sustain or improve woop?
Would you incorporate it in your work life – life outside work? *Probe for why and why not*

Discussion

- Was WOOP supported by your workplace? For example, was there enough time provided to do it and did others talk about it after the session? *Why or why not?*
- Do you think WOOP was worthwhile? *Why and why not?*

**Interaction

Part C – collective action
Was woop supported by your organisation? *Probe for why and why not*

Part D – reflexive monitoring
Do you think WOOP was worthwhile? *Probe for why and why not*

Now let's talk about the goals you set for yourself:

Discussion

- Now, think about your wish.
- In your opinion, did you attain it? If so, how? If not, why?
- What are your future intentions about your wish? What can be done to ensure you continue to use WOOP and work towards your wishes and goals in the future?

**Interaction

Think about the goals that you set (you can share your goals if you would like to but there is no need to do so)
In your opinion did you attain your goals?
How did you attain your goals? (explore enablers)
What are your future intentions about your goal? (explore continuity / sustainability of attained goal)
What can be done to ensure you continue to work on your goals in the long term?

If goals were not attained, what accounted for these? (explore barriers)
What approach(s) did you take to work on your goals?
What in your opinion accounted for the goals not being attained?
What can be done to achieve such goals in the future?

Thank you

**Interaction

Accessing more support for your health and wellbeing: Accessing more support for your health and

[redacted]

Thank you all for taking part in this discussion. [thank and close, taking any questions]
