# Supplementary material for: Protocol for a cluster randomised waitlist-controlled trial of a goal-based behaviour change intervention for employees in workplaces enrolled in health and wellbeing initiatives
Source: PLoS One. 2023 Sep 28;18(9):e0282848. doi: 10.1371/journal.pone.0282848 (PMC10538707; doi:10.1371/journal.pone.0282848)
Supplement: S3 File — a. Session 1 ‐ Part 1 –all. b. Session 1 ‐ Part 2 ‐ intervention only. c. Session 1 ‐ Part 2 ‐ control only. d. Session 2 ‐ intervention only. e. Session 2 ‐ control only. f. Handouts. (ZIP) [file pone.0282848.s003.zip › S3f. handouts.pdf]

Wish handout

Wish

Take a moment and think about the next four weeks. Consider, what is one wish or concern you have about your health and wellbeing? It does not have to be about work or your profession. Choose something that feels challenging to you but also that you feel is achievable in the next four weeks. Note your wish using no more than a few sentences:

____

____

Is this wish something that is truly important to you?

Do you believe it is possible for you to achieve your wish in four weeks?

Is it challenging for you – not too easy and not too hard?

Did you summarise it using no more than a few sentences?

Outcome

What would be the best thing – the best outcome – of fulfilling your wish? How would you feel if you were to achieve your wish?

Note your best Outcome using no more than a few sentences:

Imagine this best outcome vividly and in detail.

Obstacle

What is it within you that stops or hinders you from fulfilling your wish? What is your inner obstacle – something that you need to overcome for your wish to happen? What is it in you that is holding you back?

Note your main inner Obstacle, again using no more than a few sentences:

Imagine your main inner obstacle vividly and in detail.

Plan

What can you do to overcome your obstacle? Think about an effective action you can take, or something you can think, to overcome the obstacle. Note your action or thought using no more than a few sentences:

Now make an if-then plan.

If... ____________________________________,

then I will ____________________________________

Repeat your if-then plan and really imagine it.

Your WOOP reminder

Wish: ____

Outcome: ____________________________________

Obstacle: _____________________________________

Plan: ____

____
