# Supplementary material for: Protocol for a cluster randomised waitlist-controlled trial of a goal-based behaviour change intervention for employees in workplaces enrolled in health and wellbeing initiatives
Source: PLoS One. 2023 Sep 28;18(9):e0282848. doi: 10.1371/journal.pone.0282848 (PMC10538707; doi:10.1371/journal.pone.0282848)
Supplement: S4 File — (DOCX) [file pone.0282848.s004.docx]

# S4 –TIDieR checklist and further intervention information

**
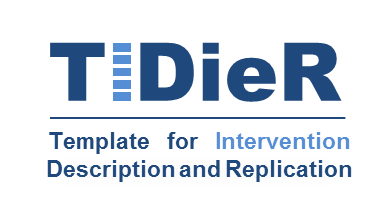
The TIDieR (Template for Intervention Description and Replication) Checklist*:**

Information to include when describing an intervention and the location of the information

| **Item number** | **Item** | **Where located **** | |
| --- | --- | --- | --- |
|  |  | Primary paper  (page or appendix  number) | Other ^†^ (details) |
|  | **BRIEF NAME** | 8 |  |
| **1.** | Provide the name or a phrase that describes the intervention. |  | _____________ |
|  | **WHY** | 3 |  |
| **2.** | Describe any rationale, theory, or goal of the elements essential to the intervention. |  | _____________ |
|  | **WHAT** | S3a-S3g |  |
| **3.** | Materials: Describe any physical or informational materials used in the intervention, including those provided to participants or used in intervention delivery or in training of intervention providers. Provide information on where the materials can be accessed (e.g. online appendix, URL). |  | _____________ |
| **4.** | Procedures: Describe each of the procedures, activities, and/or processes used in the intervention, including any enabling or support activities. | S3a-S3g | _____________ |
|  | **WHO PROVIDED** |  |  |
| **5.** | For each category of intervention provider (e.g. psychologist, nursing assistant), describe their expertise, background and any specific training given. | 8; see below | _____________ |
|  | **HOW** |  |  |
| **6.** | Describe the modes of delivery (e.g. face-to-face or by some other mechanism, such as internet or telephone) of the intervention and whether it was provided individually or in a group. | 8-9; see below | _____________ |
|  | **WHERE** |  |  |
| **7.** | Describe the type(s) of location(s) where the intervention occurred, including any necessary infrastructure or relevant features. | 8-9; see below | _____________ |
|  | **WHEN and HOW MUCH** |  |  |
| **8.** | Describe the number of times the intervention was delivered and over what period of time including the number of sessions, their schedule, and their duration, intensity or dose. | 8; see below | _____________ |
|  | **TAILORING** |  |  |
| **9.** | If the intervention was planned to be personalised, titrated or adapted, then describe what, why, when, and how. | See below | _____________ |
|  | **MODIFICATIONS** |  |  |
| **10.^ǂ^** | If the intervention was modified during the course of the study, describe the changes (what, why, when, and how). | _____________ | _____________ |
|  | **HOW WELL** |  |  |
| **11.** | Planned: If intervention adherence or fidelity was assessed, describe how and by whom, and if any strategies were used to maintain or improve fidelity, describe them. | 10-11 | To confirm that manipulation of OOP occurred as planned (fidelity), researchers delivering the sessions fill out a reporting form and one of the questions is, “Did you deliver the intervention or control session as indicated in the envelope?”_____ |
| **12.^ǂ^** | Actual: If intervention adherence or fidelity was assessed, describe the extent to which the intervention was delivered as planned. | _____________ | _____________ |

** **Authors** - use N/A if an item is not applicable for the intervention being described. **Reviewers** – use ‘?’ if information about the element is not reported/not sufficiently reported.

† If the information is not provided in the primary paper, give details of where this information is available. This may include locations such as a published protocol or other published papers (provide citation details) or a website (provide the URL).

ǂ If completing the TIDieR checklist for a protocol, these items are not relevant to the protocol and cannot be described until the study is complete.

* We strongly recommend using this checklist in conjunction with the TIDieR guide (see *BMJ* 2014;348:g1687) which contains an explanation and elaboration for each item.

* The focus of TIDieR is on reporting details of the intervention elements (and where relevant, comparison elements) of a study. Other elements and methodological features of studies are covered by other reporting statements and checklists and have not been duplicated as part of the TIDieR checklist. When a **randomised trial** is being reported, the TIDieR checklist should be used in conjunction with the CONSORT statement (see [www.consort-statement.org](http://www.consort-statement.org)) as an extension of **Item 5 of the CONSORT 2010 Statement.** When a **clinical trial** **protocol** is being reported, the TIDieR checklist should be used in conjunction with the SPIRIT statement as an extension of **Item 11 of the SPIRIT 2013 Statement** (see [www.spirit-statement.org](http://www.spirit-statement.org)). For alternate study designs, TIDieR can be used in conjunction with the appropriate checklist for that study design (see [www.equator-network.org](http://www.equator-network.org)).

**Further intervention information**

*What.* Participants will attend a group session with a researcher to discuss health and wellbeing at work. The session includes data collection and/or intervention activities. The intervention is available PowerPoint format, which can be delivered virtually or in person. Participants will also be provided with a booklet to aid their engagement and completion of the study (see Appendix 3 for PowerPoint and booklet). The booklet contains prompts to complete the intervention.

Immediately following baseline survey data collection, researchers will open an envelope to determine if the intervention will be delivered to their group during the session. If the group are assigned to the intervention, the facilitator will proceed to introduce them to the mental contrasting and implementation intentions intervention. This introduction includes how mental contrasting may benefit the individual giving examples from previous studies. The information also details mental contrasting in the form of a memorable acronym to encourage compliance with the intervention. The ‘WOOP’ (Wish, Outcome, Obstacle, Plan) acronym is a consumable method of performing mental contrasting and implementation intentions. The participants are introduced to the concepts within ‘WOOP’ and then to increase familiarity, the participants are asked to perform a ‘WOOP’.

Participants are given their booklet (S3) to takeaway and encouraged to use this to complete the intervention. To close the session participants are asked if they have any questions regarding the study or intervention. Participants are also shown a ‘frequently asked questions’ page which may attend to any questions. Participants are provided with contact details of the research team and a health and wellbeing champion in case they have any questions at a later date.

*Who provided.* A member of the research team (MSc+) familiar with literature and use of mental contrasting, implementation intentions, and WOOP interventions will provide the sessions. They will have PowerPoint slides and some text to follow during the session. All members of the research team running the sessions will watch videos and listen to audios from the WOOP website (woopmylife.org), attend a training session run by [blind for peer review], and pilot the sessions – receiving feedback from other researchers.

*How.* At the discretion of the organisation enrolled on the study, they will select whether session delivery will be virtual (Zoom, Microsoft Teams or another platform) or in person, according to convenience and what they believe will maximise uptake. materials can be used digitally or provided as hard copies. Also at the discretion of the organisation is whether the session is delivered solely by a member of the research team or supported by a local health and wellbeing champion, depending on convenience and what they believe will maximise uptake.

*Where.* When conducted virtually, the intervention will be conducted on Zoom, Microsoft teams, or similar. If the intervention session is being delivered in person, a meeting space within the workplace will be used.

*When and how much.* The intervention is delivered once in a group setting lasting around one hour.

*Tailoring.* Whilst the aim is to have consistency in delivery across all facilitators with the PowerPoint slides and suggested text, it is also expected and encouraged that the facilitators will deploy their own delivery style, energy and enthusiasm according to their audience to maximise engagement and uptake from participants. Examples of ‘woops’ in the PowerPoint slides are unique to the facilitators and could be modified in the future by other facilitators. If health and wellbeing champions support the delivery, they will also have their own approach. All facilitators will have suggested text to aid their delivery and local health and wellbeing champions are required to attend an information session organised by the research team prior to the group session with employees.
