# Supplementary figures and images for: Protocol for a cluster randomised waitlist-controlled trial of a goal-based behaviour change intervention for employees in workplaces enrolled in health and wellbeing initiatives
Source: PLoS One. 2023 Sep 28;18(9):e0282848. doi: 10.1371/journal.pone.0282848 (PMC10538707; doi:10.1371/journal.pone.0282848)

Figure S5: Causal chain from intervention to outcomes


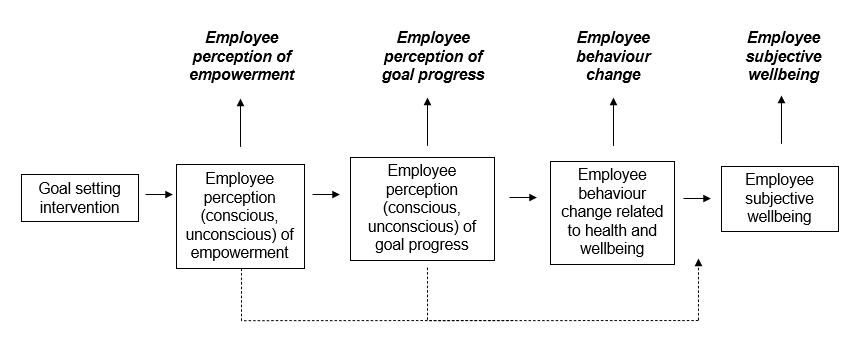

Supplement: S5 File — (DOCX) [file pone.0282848.s005.docx]
