# Supplementary material for: Protocol for a cluster randomised waitlist-controlled trial of a goal-based behaviour change intervention for employees in workplaces enrolled in health and wellbeing initiatives
Source: PLoS One. 2023 Sep 28;18(9):e0282848. doi: 10.1371/journal.pone.0282848 (PMC10538707; doi:10.1371/journal.pone.0282848)
Supplement: S6 File — (DOCX) [file pone.0282848.s006.docx]

# S6 – Semi-structured focus group discussion guide

**Group discussion on mechanisms, barriers, enablers (endpoint, intervention group 1 only)**

I would like us to take some time to talk about wish, outcome, obstacle and plan, also called ‘woop’ or ‘mental contrasting with implementation intentions’ [if needed, recall this is something that we practiced several weeks ago, which was about how to think about the future and any obstacles related to what you wish to do for your health and wellbeing]

*Part 1 – Normalisation process theory items on mechanisms*

*Part A - coherence*

What do you think is the difference between woop and what is offered at work?

What was the purpose of woop and what was required of you when taking part?

How would/did woop affect/influence your health and wellbeing?

*Part B - cognitive participation*

What will help sustain or improve woop?

Would you incorporate it in your work life? *Probe for why and why not*

*Part C – collective action*

Was woop supported by your organisation? *Probe for why and why not*

*Part D – reflexive monitoring*

Do you think WOOP was worthwhile? *Probe for why and why not*

Now let’s talk about the goals you set for yourself:

Part 2 - analytic themes about barriers and enablers

Think about the goals that you set (you can share your goals if you would like to but there is no need to do so)

In your opinion did you attain your goals?

How did you attain your goals? (explore enablers)

What are your future intentions about your goal? (explore continuity / sustainability of attained goal)

What can be done to ensure you continue to work on your goals in the long term?

If goals were not attained, what accounted for these? (explore barriers)

What approach(s) did you take to work on your goals?

What in your opinion accounted for the goals not being attained?

What can be done to achieve such goals in the future?

Thank you all for taking part in this discussion.
