# Supplementary material for: Protocol for a cluster randomised waitlist-controlled trial of a goal-based behaviour change intervention for employees in workplaces enrolled in health and wellbeing initiatives
Source: PLoS One. 2023 Sep 28;18(9):e0282848. doi: 10.1371/journal.pone.0282848 (PMC10538707; doi:10.1371/journal.pone.0282848)
Supplement: S7 File — (DOCX) [file pone.0282848.s007.docx]

# S7 – Brief summary timeline

| *Trial activities* | May | Jun | Jul | Aug | Sep | Oct | Nov | Dec | Jan | Feb | Mar |
| --- | --- | --- | --- | --- | --- | --- | --- | --- | --- | --- | --- |
| Invite workplaces to sessions (organisational enrolment) | …………………………………………………………… | | | | | | | | | |  |
|  |  |  |  |  |  |  |  |  |  |  |  |
| Randomisation of workplaces |  |  |  |  | ………………………………… | | | | | |  |
|  |  |  |  |  |  |  |  |  |  |  |  |
| Intervention Group 1 – Sessions | | | | | | | | | | | |
| Session 1 (participant recruitment and consent, baseline data collection from participants, group intervention) |  |  |  |  |  | …………………………… | | | | |  |
| Session 2 (4+ weeks later - endline data collection from participants) |  |  |  |  |  | | …………………………… | | | | |
| Control Group 2 – Sessions | | | | | | | | | | | |
| Session 1 (participant recruitment and consent, baseline data collection from participants, no intervention) |  |  |  |  |  | …………………………… | | | | |  |
| Session 2 (4+ weeks later - endline data collection, group intervention) |  |  |  |  |  |  | …………………………… | | | | |
