# Supplementary material for: Protocol for a cluster randomised waitlist-controlled trial of a goal-based behaviour change intervention for employees in workplaces enrolled in health and wellbeing initiatives
Source: PLoS One. 2023 Sep 28;18(9):e0282848. doi: 10.1371/journal.pone.0282848 (PMC10538707; doi:10.1371/journal.pone.0282848)
Supplement: S8 File — (DOCX) [file pone.0282848.s008.docx]

# S8 - Organisational information sheet and commitment

**Organisational Information Sheet**

**Project title: A study of workplace health and wellbeing programmes in England**

The longer academic titles that you may see written in other places are, “A mixed-methods evaluation of cross-regional workplace health initiatives including a cluster randomised controlled trial (cRCT) of a behaviour change intervention” and “A cluster randomised waitlist-controlled trial of a goal-setting behaviour change intervention for employees in workplaces enrolled in workplace health and wellbeing initiatives.”

Your organisation is invited to take part in a research study. Before you decide, you need to understand why the study is being done and what it would involve for you. Please take the time to read the following information carefully. Please contact [anonymous for peer review] if there is anything that is not clear or if you would like more information.

**What is this project about?**

This project is about workplace health and wellbeing. We want to find out if there are ways to improve people’s health and wellbeing by better understanding what happens to them at work. We are talking to people and analysing questions from surveys. To evaluate the effectiveness of efforts to improve workplace health and wellbeing, we would like to talk to employees of organisations like yours that have workplace health and wellbeing initiatives.

**Does my organisation have to take part?**

No, your organisation does not have to take part. You will not be penalised if you do not take part. It is entirely up to you to decide.

**What will happen if I decide that my organisation will take part?**

A researcher will contact you to arrange contact with employees at your organisation who can decide themselves if they want to participate in the research study. The researcher will be from [anonymous for peer review]. Employees at your organisation will be asked if they would be willing to take part two group discussions about health and wellbeing at work lasting one hour each. The first discussion would be in August or September and the second discussion would be in October or November 2022. The discussion could be in person or virtual depending on what you think is best.

Staff would be asked some questions about who you are, where you work and your experience with health and wellbeing at work. Some of the organisations involved in the research will receive extra help implementing content about health and wellbeing at work.

If staff at you organisation choose to participate, they will be asked to sign a consent form to confirm that they have agreed to take part. They will be free to withdraw at any time before 31 March 2023, without giving a reason, and this will not affect them or their circumstances in any way. If choosing to withdraw from the study, any data collected before withdrawal will be deleted and quotes will be removed from any written reports if it is possible to do so. However, after 31 March 2023, this may not be possible because publication may have already taken place.

We will ask that focus group participants keep each other’s identities/contributions confidential outside the group.

**Who is organising and funding the study?**

The study is funded by [anonymous for peer review]. More information about the funding is available online here: [anonymous for peer review] There are researchers from the [anonymous for peer review]. The project has received ethics approval from [anonymous for peer review].

**Why was my organisation asked to take part in the project?**

Your organisation was asked to take part in the study because your organisation participates in [anonymous for peer review]. We would like to understand your experience of health and wellbeing at work and people in your organisation some questions about it.

**What are the dates for the research?**

Research for this part of the project will take place between August and December 2022.

**How long will the interviews last?**

The online discussions are expected to last around 45 minutes to one hour.

**Will personal information be kept private?**

Yes, all information about staff will be kept very safe and private. Names will not be used in any reports so no one will know what they have said. If someone says something that makes us think that they or anyone else may be at risk of harm, then we may have to speak to someone outside of the research team. Discussions may be transcribed by computer software on Zoom or Microsoft Teams or a professional transcription company. These transcriptions will be password-protected. Direct quotes may be used in reports, but this will not include any identifiable characteristics (quotes will be anonymised).

If anyone reports anything to us that suggests risk of harm, we may need to report our safeguarding concerns to [anonymous for peer review]

**What will happen to the data?**

Password protected files stored on secure [anonymous for peer review] servers will contain names, discussion dates and times, and ID codes. This document will be stored separately from the data on questions people answer. The questions people answer will be stored on password-protected Cloud storage associated with a [anonymous for peer review], or handheld recording devices if you consent to be recorded in a discussion.

Answers to the questions will be downloaded and saved by ID code into a password-protected folder on secure [anonymous for peer review] storage. After the data are downloaded from the Cloud, data stored on the Cloud will be deleted.

The data may be pseudo-anonymised and shared between [anonymous for peer review]. When data are pseudo-anonymised, it means that names are removed along with any other information that could identify people, such as where they work or live ([anonymous for peer review]). The data are pseudo-anonymised and not anonymised because the ID code of your interview could still link responses to names if somebody had the Excel document containing both the name and ID code. However, the Excel document with names and ID codes will only be accessible by approved [anonymous for peer review] researchers.

None of the responses will be attributable to named individuals in all publications and reports. At the end of the projects, the document linking names to ID codes will be deleted (31 December 2023). The anonymised data will be stored for 10 years according to [anonymous for peer review].

**What are the benefits and risks associated with taking part in this research?**

Sometimes people benefit from taking part in research like this because they pay more attention to their own health and wellbeing as a result, which can improve it. It is an opportunity to contribute to improving our understanding of health and wellbeing, which may benefit you and others if this knowledge is successfully applied later on to improve health and wellbeing. There are minimal risks involved with your participation in this research, although we will ask for some of your time that you could spend doing other things.

If you are experiencing mistreatment at work, you can contact [anonymous for peer review].

**Can I find out the results of the research?**

Yes. After we finish collecting and analysing the data we will write publications and reports about what we have found and share them so that other people can use them to better understand and improve individual and community wellbeing. A summary of the findings in non-technical language will be available at the end of the project and is available upon request from [anonymous for peer review]

**Who can I contact if I have questions?**

If you have any questions or concerns regarding the research then please do not hesitate to contact [anonymous for peer review].

**Workplace Health and Wellbeing - Organisational Commitment**

I commit to supporting the research on workplace health and wellbeing in my organisation by:

• speaking to a researcher about the study in May ‘22 – Mar ‘23

• helping to arrange contact with employees who may volunteer to participate in two one hour group sessions either virtually or in person - the first in Oct ’22 – Feb ‘23 of 2022, and the second in Nov ‘22 – Mar ‘23

____________________________________________________Name

____________________________________________________Name of organisation

____________________________________________________Signature

____________________________________________________Date

**What is the best way to contact you initially and to keep in contact with you as needed over time?**

**____________________________________________________**

**Approximately how many employees work in your organisation (best estimate)?**

Less than 10, 10-50, 51-100, 101-499, 500-1000, 1000-5000, 5000-10,000, 10,000+

**What is the nature of your organisation's business?**

Manufacturing / commercial / manual (including water supply, waste management, manufacturing, construction, transportation, etc); services (including IT, finance, insurance, real estate, accommodation, food, etc); social / public / intellectual (including social enterprise, education, arts, entertainment, etc)

Other (please specify) ________________________________________________

No answer / don't know
