# Supplementary material for: Protocol for a cluster randomised waitlist-controlled trial of a goal-based behaviour change intervention for employees in workplaces enrolled in health and wellbeing initiatives
Source: PLoS One. 2023 Sep 28;18(9):e0282848. doi: 10.1371/journal.pone.0282848 (PMC10538707; doi:10.1371/journal.pone.0282848)
Supplement: S9 File — (DOCX) [file pone.0282848.s009.docx]

# S9 - Quantitative survey questionnaire

Intro

Thank you for agreeing to take part in this research about your perceptions of health and wellbeing. To confirm, your individual responses will not be shared with other people in your workplace. Your name will not be used in any reports so no one will know what you have said. First, we have a few questions about your wellbeing.

Subjective wellbeing [Random order]

1. Overall, how satisfied are you with your life nowadays?
2. Overall, to what extent do you feel the things you do in your life are worthwhile?
3. Overall, how happy did you feel yesterday?
4. Overall, how anxious did you feel yesterday? (for this question only, 0 is better wellbeing [i.e not experiencing anxiety] and 10 is worse wellbeing [i.e experiencing high anxiety])
5. Overall, how satisfied are you with your job nowadays?
6. How meaningful do the activities that you do at work feel to you?
7. How enjoyable do the activities that you do at work feel to you?

All 0-10 scale, not at all to completely. Items 1-4 are the UK’s Office for National Statistics four items used to monitor national wellbeing [1,2].^[[1]](#footnote-2)^ Item 5 is a common measure of job satisfaction [3].^[[2]](#footnote-3)^ Items 6-7 are adapted from literature on meaningful work [4].^[[3]](#footnote-4)^

Mental wellbeing – SWEMWBS

1. I’ve been feeling optimistic about the future
2. I’ve been feeling relaxed
3. I’ve been dealing with problems well
4. I’ve been thinking clearly
5. I’ve been feeling close to other people
6. I’ve been able to make up my own mind about things

All 1-5 scale, none of the time, rarely, some of the time, often, all of the time. Items 8-9 are from SWEMWBS, a short-form measure of mental wellbeing [5].^[[4]](#footnote-5)^

Empowerment attitudes, knowledge, and behaviours

1. I am confident in my ability to look after my health and wellbeing
2. I know what to do to improve my health and wellbeing
3. When problems arise with my health and wellbeing, I handle them well
4. I am confident that I can make the best choices to look after my health and wellbeing
5. How much do you agree with the statement, “I feel physically safe at work?”

All 1-5 scale, strongly disagree to strongly agree. Items 14-17 are adapted from prior empowerment scales [6-9].^[[5]](#footnote-6)^ Item 18 has been used in prior research [10].^[[6]](#footnote-7)^

_________________________________________

*Endpoint only (assess progress on goal/wish):*

1. To confirm, is this your second session with us? (Yes/No/Unsure) - if Yes/Unsure, thank and close
2. Before the last session, had you ever heard of WOOP (Wish, Outcome, Obstacle, Plan) or ‘Mental Contrasting plus Implementation Intentions?’ Yes/No/Unsure
3. As a reminder, in the previous session, you may have been asked to tell us about something that you wished to do for your health and wellbeing. Do you remember being asked about this? Yes/No/Unsure

As a reminder: It was supposed to be something challenging, but feasible to achieve in around four weeks. It did not need to be work-related, but it should have been something that you thought that you could complete in around four weeks.

1. What did you wish to do for your health and wellbeing? (if you do not remember, leave this blank). __________________________________
2. So far, how much progress would you say that you have made towards what you wished to do for your health and wellbeing? No progress – a lot of progress (1-7) OR don’t remember what I wished to do
3. How much progress in *changing your behaviour* would you say you have made towards what you wished to do for your health and wellbeing?” No progress – a lot of progress (1-7) OR don’t remember what I wished to do
4. What was your wish about? Health, social relationships, work performance, academic, other

Items 19-22 and 25 are based on prior literature [11-13].^[[7]](#footnote-8)^ Items 23-24 are adapted from prior research [14].^[[8]](#footnote-9)^

Now we have a few questions about you and where you work.

1. Are you a Thrive at Work lead for your organisation? Yes/No/Unsure

Item 26 refers to the local government health and wellbeing programme.^[[9]](#footnote-10)^

1. What is your sex? (male, female, intersex, other specify)
2. Is the gender you identify with the same as your sex registered at birth? (yes, no - write in gender identity)
3. What year were you born?
4. What is your ethnicity? (White; Mixed or Multiple Ethnic Groups; Asian or Asian British; Black, Black British, Caribbean or African, Other, Prefer not to state)
5. Do you have any physical or mental health conditions or illnesses lasting or expected to last 12 months or more? If yes please expand.
6. How is your health in general? (very good, good, fair, bad, very bad)

Items 27-32 come from the UK 2021 official Census questionnaire.^[[10]](#footnote-11)^

1. In the last six months, how many times have you made an appointment with a GP or hospital about a health problem that you experienced? (0-10+)

Item 33 is asked to assess healthcare access and utilisation and is not adapted from a specific prior questionnaire.

1. Which group does your job belong to? Please select one:

Managers

Professionals

Technicians and Associate Professionals

Clerical Support Workers

Services and Sales Workers

Skilled Agricultural, Forestry and Fishery Workers

Craft and Related Trades Workers

Plant and Machine Operators and Assemblers

Elementary Occupations

Armed Forces Occupations

Other (Write in: _____________)

Item 34 comes from the International Standard Classification of Occupations [15].

1. Did you do any working from home in the last week? Yes/No

Item 35 comes from the UK Office for National Statistics [16].

1. How long have you been working for your organisation? (Less than 2 years, 2 to less than 5 years, 5 to less than 10 years, 10 years or more)

Item 36 is a general measure of job duration and is not adapted from a specific prior questionnaire.

1. Have you achieved a qualification at degree level or above? For example, degree, foundation degree, HND or HNC, NVQ level 4 and above, teaching or nursing.

Items 37 comes from the UK 2021 official Census questionnaire.^[[11]](#footnote-12)^

1. Finally, what is your annual household income (your best estimate, after taxes)? As a reminder, we are asking this question to find out if workplace initiatives like this work better for some people than for others. Your replies will not be shared with other people in your workplace and will be reported separately to your name. This is an optional question. Less than 10K, 10-20K, 20-30K, 30-40K, 40-50K, 50-60K, 60-70K, 70-80K, 80K+

Item 38 was selected to align with reporting from the UK’s Office for National Statistics on average disposable household income: https://www.ons.gov.uk/peoplepopulationandcommunity/personalandhouseholdfinances/incomeandwealth/bulletins/householddisposableincomeandinequality/financialyearending2021

**References**

1. Office for National Statistics. Personal wellbeing user guidance. https://www.ons.gov.uk/peoplepopulationandcommunity/wellbeing/methodologies/personalwellbeingsurveyuserguide
2. Hicks S, Tinkler L, Allin P. Measuring subjective well-being and its potential role in policy: Perspectives from the UK Office for National Statistics. Social Indicators Research. 2013 Oct;114(1):73-86.
3. Clark A. Pioneer in Subjective Quality of Life Research: Andrew E. Clark. Applied Research in Quality of Life. 2022 Apr;17(2):1181-4.
4. Martikainen SJ, Kudrna L, Dolan P. Moments of meaningfulness and meaninglessness: a qualitative inquiry into affective eudaimonia at work. Group & Organization Management. 2021 Dec 1:10596011211047324.
5. Stewart-Brown S, Tennant A, Tennant R, Platt S, Parkinson J, Weich S. Internal construct validity of the Warwick-Edinburgh Mental Well-Being Scale (WEMWBS): A Rasch analysis using data from the Scottish Health Education Population Survey. Health Qual Life Outcomes. 2009;7: 15. doi:10.1186/1477-7525-7-15.
6. Koren PE, DeChillo N, Friesen BJ. Measuring empowerment in families whose children have emotional disabilities: A brief questionnaire. Rehabil Psychol. 1992;37: 305–321. doi:10.1037/h0079106
7. Segers EW, van den Hoogen A, van Eerden IC, Hafsteinsdóttir T, Ketelaar M. Perspectives of parents and nurses on the content validity of the Family Empowerment Scale for parents of children with a chronic condition: A mixed‐methods study. Child: Care, Health and Development. 2019 Jan;45(1):111-20.
8. Azcurra DJLS. Escala de Empoderamiento sobre la Salud para Adultos Mayores. Adaptación al español y análisis psicométrico. Colomb Med. 2014;45: 179–185. Available: /pmc/articles/PMC4350384/
9. Anderson, R.M., Fitzgerald, J.T., Gruppen, L.D., Funnell, M.M. and Oh, M.S., 2003. The diabetes empowerment scale-short form (DES-SF). Diabetes Care, 26(5), pp.1641-1642.
10. Peters SE, Sorensen G, Katz JN, Gundersen DA, Wagner GR. Thriving from work: conceptualization and measurement. International Journal of Environmental Research and Public Health. 2021 Jul 5;18(13):7196.
11. Schweiger Gallo I, Bieleke M, Alonso MA, Gollwitzer PM, Oettingen G. Downregulation of anger by mental contrasting with implementation intentions (MCII). Frontiers in Psychology. 2018 Oct 4;9:1838.
12. Von Weichs V, Krott NR, Oettingen G. The Self-Regulation of Conformity: Mental Contrasting With Implementation Intentions (MCII). Frontiers in Psychology. 2021 Jun 2;12:546178.
13. Gollwitzer PM, Mayer D, Frick C, Oettingen G. Promoting the self-regulation of stress in health care providers: An internet-based intervention. Frontiers in Psychology. 2018 Jun 15;9:838.
14. Chen P, Powers JT, Katragadda KR, Cohen GL, Dweck CS. A strategic mindset: An orientation toward strategic behavior during goal pursuit. Proceedings of the National Academy of Sciences. 2020 Jun 23;117(25):14066-72.
15. The International Standard Classification of Occupations -ISCO-08. https://isco-ilo.netlify.app/en/isco-08/
16. Office for National Statistics. Employment and Labour Market. ]https://www.ons.gov.uk/employmentandlabourmarket/peopleinwork/employmentandemployeetypes/bulletins/coronavirusandhomeworkingintheuk/april2020#measuring-the-data

1. Reference [2] in *Social Indicators Research* publishes under CC BY (https://www.springer.com/journal/11205/how-to-publish-with-us) [↑](#footnote-ref-2)
2. Reference [3] in *Applied Research in Quality of Life* publishes under CC BY (https://www.springer.com/journal/11482/how-to-publish-with-us). [↑](#footnote-ref-3)
3. Reference [4] has a CC BY license: https://research.birmingham.ac.uk/en/publications/moments-of-meaningfulness-and-meaninglessness-a-qualitative-inqui [↑](#footnote-ref-4)
4. Reference [5] has a CC BY license: https://hqlo.biomedcentral.com/submission-guidelines/copyright [↑](#footnote-ref-5)
5. Reference [6] has a CC BY license (<https://www.ncbi.nlm.nih.gov/pmc/articles/PMC7380029/>) [↑](#footnote-ref-6)
6. Reference [10] has a CC BY license (<https://www.mdpi.com/1660-4601/18/13/7196>) [↑](#footnote-ref-7)
7. References [11-13] in *Frontiers in Psychology* are published under a CC-BY license (https://www.frontiersin.org/legal/copyright-statement) [↑](#footnote-ref-8)
8. Reference [14] states it has a CC BY license, “This open access article is distributed under Creative Commons Attribution-Non Commercial-No Derivatives License 4.0 (CC BY-NC-ND).” [↑](#footnote-ref-9)
9. https://www.wmca.org.uk/what-we-do/thrive/thrive-at-work/ [↑](#footnote-ref-10)
10. https://www.ons.gov.uk/census/censustransformationprogramme/questiondevelopment/census2021paperquestionnaires [↑](#footnote-ref-11)
11. https://www.ons.gov.uk/census/censustransformationprogramme/questiondevelopment/census2021paperquestionnaires [↑](#footnote-ref-12)
