# Supplementary material for: Protocol for a cluster randomised waitlist-controlled trial of a goal-based behaviour change intervention for employees in workplaces enrolled in health and wellbeing initiatives
Source: PLoS One. 2023 Sep 28;18(9):e0282848. doi: 10.1371/journal.pone.0282848 (PMC10538707; doi:10.1371/journal.pone.0282848)
Supplement: S10 File — (DOCX) [file pone.0282848.s010.docx]

**Workplace Health and Wellbeing - Participant Information Sheet**

You are invited to take part in a research study. The research study involves survey questionnaires and some questions we will ask you and your colleagues in a group discussion about the session. Before you decide if you want to participate in the research, you need to understand why the study is being done and what it would involve for you. Please take the time to read the following information carefully. Please contact [anonymous for peer review] if there is anything that is not clear or if you would like more information.

**Who is running the project?**

Researchers at [anonymous for peer review] are running the project.

**What is this project about?**

This project is about workplace health and wellbeing. We want to find out if there are ways to improve people’s health and wellbeing by better understanding what happens to them at work. We are talking to people and analysing questions from surveys. To evaluate the effectiveness of efforts to improve workplace health and wellbeing, we would like to talk to employees of organisations like yours that have workplace health and wellbeing initiatives.

**What is the project called?**

# The project is called, ‘A study of workplace health and wellbeing programmes in England.’ The longer academic titles that you may see written in other places are, “A mixed-methods evaluation of cross-regional workplace health initiatives including a cluster randomised controlled trial (cRCT) of a behaviour change intervention” and “A cluster randomised waitlist-controlled trial of a goal-setting behaviour change intervention for employees in workplaces enrolled in workplace health and wellbeing initiatives.”

**Do I have to take part?**

No, you do not have to take part. You will not be penalised if you do not take part. It is entirely up to you to decide. You can still take part in the goal-setting sessions, but please let us know if you decide not to take the survey or do not want your replies to our questions to be used.

**What will happen to me if I take part?**

A researcher will contact you to arrange a research activity. The researcher will be from the [anonymous for peer review]. You could be asked to take part in:

- **A survey questionnaire**. The surveys have around 10-20 minutes of questions that can be answered online, over the phone, or on paper in person. You might be asked to click on a link that is emailed to you or provided to you during an online session. You could be asked some questions on the phone, or you could be given a paper survey.
- **A group discussion**. You and your colleagues would be invited to have a discussion with a researcher in a ‘focus group’ session that involves a group discussion over Zoom or Microsoft Teams or in person, which should last around for around 40 minutes.

You will be asked some questions about who you are, where you work and your experience with health and wellbeing at work. All of the organisations involved in the research will receive extra help implementing content about health and wellbeing at work.

If you choose to participate, we will ask you to sign a consent form to confirm that you have agreed to take part. You will be free to withdraw at any time, without giving a reason, and this will not affect you or your circumstances in any way. If choosing to withdraw from the study, any data collected before withdrawal will be deleted and quotes will be removed from any written reports if it is possible to do so. However, after 31 March 2023, this may not be possible because publication may have already taken place. If withdrawing from the group discussion, anything said in the group discussion prior to the decision to leave cannot be withdrawn given the nature of group discussions.

We ask that participants in the online discussion keep each other’s identities and contributions confidential outside the group.

**Who is organising and funding the study?**

The study is funded by [anonymous for peer review]. There are researchers from [anonymous for peer review]. The project has received ethics approval from [anonymous for peer review].

**Why was I asked to take part in the project?**

You were asked to take part in the study because people who work at your organisation have said they are interested in workplace health and wellbeing. We would like to understand your experience of health and wellbeing at work and ask you some questions about it.

**What are the dates for the research?**

Interviews will take place between September 2021 and March 2023.

**How long will the research last?**

The online surveys are expected to last around 10-20 minutes and the online discussions are expected to last around 20-30 minutes (depending on people’s answers).

**Will my personal information be kept private?**

Yes, all information about you will be kept very safe and private. Your name will not be used in any reports so no one will know what you have said. If you say anything that makes us think that you or anyone else may be at risk of harm, then we may have to speak to someone outside of the research team. Your interviews may be transcribed by computer software on Zoom or Microsoft Teams or a professional transcription company. These transcriptions will be password-protected. Direct quotes may be used in reports but this will not include any identifiable characteristics (quotes will be anonymised).

If you report anything to us that suggests you are at risk of harming yourself or others we may need to report our safeguarding concerns to [anonymous for peer review].

**What will happen to my data?**

Password protected files stored on secure University servers will contain your name, interview date and time, and ID code. This document will be stored separately from the data on questions you answer. The questions you answer will be stored on password-protected Cloud storage associated with a University, or handheld recording devices if you consent to be recorded in a discussion.

Answers to your questions will be downloaded and saved by ID code into a password-protected folder on secure University storage. After your data are downloaded from the Cloud, data stored on the Cloud will be deleted.

Your data may be pseudo-anonymised and shared between [anonymous for peer review]. When data are pseudo-anonymised, it means that your name is removed along with any other information that could identify you, such as where you work or live (https://ico.org.uk/for-organisations/guide-to-data-protection/guide-to-the-general-data-protection-regulation-gdpr/key-definitions/what-is-personal-data/). The data are pseudo-anonymised and not anonymised because the ID code of your interview could still link your responses to your name if somebody had the Excel document containing your name and ID code. However, the Excel document with your name and ID code will only be accessible by approved University researchers.

None of your responses will be attributable to you in all publications and reports. At the end of the projects, the document linking your name to your ID code will be deleted (31 December 2023). Your anonymised data will be stored for 10 years according to the [anonymous for peer review] guidelines.

**What are the benefits and risks associated with taking part in this research?**

Sometimes people benefit from taking part in research like this because they pay more attention to their own health and wellbeing as a result, which can improve it. It is an opportunity to contribute to improving our understanding of health and wellbeing, which may benefit you and others if this knowledge is successfully applied later on to improve health and wellbeing. There are minimal risks involved with your participation in this research, although we will ask for some of your time that you could spend doing other things.

If you are experiencing mistreatment at work, you can contact [anonymous for peer review].

**Can I find out the results of the research?**

Yes. After we finish collecting and analysing the data we will write publications and reports about what we have found and share them so that other people can use them to better understand and improve individual and community wellbeing. A summary of the findings in non-technical language will be available at the end of the project and is available upon request from [anonymous for peer review].

**Who can I contact if I have questions?**

If you have any questions or concerns regarding the research then please do not hesitate to contact [anonymous for peer review]. If you have any concerns/complains about the research process you can also contact [anonymous for peer review].

**Project: Workplace Health and Wellbeing - Consent Form**

Please read the statements below and complete the form by adding a ‘**X’** if you want to answer ‘Yes’ or leave blank if your answer is ‘No’ for each of the statements below.

1. **I consent to take part in:  for yes  for no (select all that apply)**

The survey questionnaires

The group discussion

1. **I consent to:  for yes  for no (select all that apply)**

The discussion being audio and video recorded

1. **I confirm that:  for yes  for no**

I have read and understand the participant information leaflet (Participant Information Sheet) for this study*

I have had the opportunity to ask questions and receive satisfactory answers*

I understand that my participation is voluntary and that I am free to withdraw at any time without giving any reason*

I understand that if I withdraw my interview data before 31 May 2023 it will be removed from the study and will be destroyed*

I understand that if I withdraw from the group discussion that I am unable to withdraw anything I have said in the group discussion prior to my decision to leave given the nature of group discussions*

I understand that my personal data will be processed for the purposes detailed above, in accordance with the [anonymous for peer review] and Data Protection Act 2018*

I understand that my pseudo-anonymous data (this means that your name is removed along with any other information that could identify you) may be looked at by staff [anonymous for peer review].

I understand that discussion group recordings may be sent to an external company for transcription*

I understand that my quotes may be used in reports and publications, but these will not include any identifiable characteristics (quotes will be anonymised)*

Based upon the above, I agree to take part in this study*

*Required to proceed to survey or group discussion

Please write your first and surname to confirm your consent and attendance (this is only for consent and attendance purposes, your replies will remain confidential and your name will not be reported with them)

Name: ……………………………….

Work email address (if you have one): ……………………………….

Date………………………

Project - A study of workplace health and wellbeing programmes in England. This information is being collected as part of a research project concerned with workplace health and wellbeing by a collaboration of researchers from the [anonymous for peer review]. The information which you supply and that which may be collected as part of the research project will be entered into a filing system or database and will only be accessed by authorised personnel involved in the project. The information will be retained by the University and will only be used for the purpose of research, and statistical and audit purposes. By supplying this information you are consenting to the University storing your information for the purposes stated above. The information will be processed by the University in accordance with the provisions of the GDPR and Data Protection Act 2018. No identifiable personal data will be published.
