# Supplementary material for: Protocol for a cluster randomised waitlist-controlled trial of a goal-based behaviour change intervention for employees in workplaces enrolled in health and wellbeing initiatives
Source: PLoS One. 2023 Sep 28;18(9):e0282848. doi: 10.1371/journal.pone.0282848 (PMC10538707; doi:10.1371/journal.pone.0282848)
Supplement: S11 File — (DOCX) [file pone.0282848.s011.docx]

# S11 – Debriefing information for workplaces

**Workplace Health and Wellbeing - Participant Information Sheet – Further Information**

# Project title: A study of workplace health and wellbeing programmes in England

**Investigator (s): Investigator [anonymous for peer review]**

Recently you may have participated in an activity about your health and wellbeing for [anonymous for peer review]. In the activity, people were asked to set a goal and think about how they could go about achieving and accomplishing the goal. As part of the activity, researchers were testing whether setting goals in this way improves the chances of success and wellbeing. Thank you for taking part.

Would you like further information? Email the Project Investigator [anonymous for peer review]
