# Supplementary material for: Protocol for a cluster randomised waitlist-controlled trial of a goal-based behaviour change intervention for employees in workplaces enrolled in health and wellbeing initiatives
Source: PLoS One. 2023 Sep 28;18(9):e0282848. doi: 10.1371/journal.pone.0282848 (PMC10538707; doi:10.1371/journal.pone.0282848)
Supplement: S12 File — (ZIP) [file pone.0282848.s012.zip › aer_application for ethical review_ v5.docx]

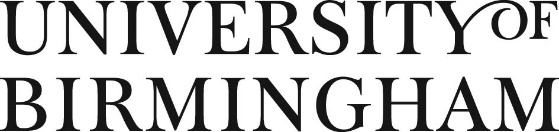


**Application for Ethics Review Form**

# Guidance Notes:

**What is the purpose of this form?**
This form should be completed to seek ethics review for research projects to be undertaken by University of Birmingham staff, PGR students or visiting/emeritus researchers who will be carrying out research which will be attributed to the University.

**Who should complete it?**
For a staff project – the lead researcher/Principal Investigator on the project.
For a PGR student project – the student’s academic supervisor, in discussion with the student.

Students undertaking undergraduate projects and taught postgraduate (PGT) students should refer to their Department/School for advice

**When should it be completed?**
After you have completed the University’s online ethics self-assessment form (SAF), **IF** the SAF indicates that ethics review is required. You should apply in good time to ensure that you receive a favourable ethics opinion prior to the commencement of the project and it is recommended that you allow at least 60 working days for the ethics process to be completed.

**How should it be submitted?**
An electronic version of the completed form should be submitted to the Research Ethics Officer, at the following email address: aer-ethics@contacts.bham.ac.uk.

**What should be included with it?**
Copies of any relevant supporting information and participant documentation, research tools (e.g. interview topic guides, questionnaires, etc) and where appropriate a health & safety risk assessment for the project (see section 10 of this form for further information about risk assessments).

**What should applicants read before submitting this form?**
Before submitting, you should ensure that you have read and understood the following information and guidance and that you have taken it into account when completing your application:

- The information and guidance provided on the University’s ethics webpages (<https://intranet.birmingham.ac.uk/finance/accounting/Research-Support-Group/Research-Ethics/Ethical-Review-of-Research.aspx>)
- The University’s Code of Practice for Research (<https://www.birmingham.ac.uk/Documents/university/legal/research.pdf>)
- The guidance on Data Protection for researchers provided by the University’s Legal Services team at  [https://intranet.birmingham.ac.uk/legal-services/What-we-do/Data-Protection/resources.aspx](https://mail.bham.ac.uk/owa/redir.aspx?C=LTQ9IWbUuWQM_lucrdpGKwuM9bGwzORQSLFN7aSQvhi3N_JHBLLWCA..&URL=https%3a%2f%2fintranet.birmingham.ac.uk%2flegal-services%2fWhat-we-do%2fData-Protection%2fresources.aspx).

# Section 1: Basic Project Details

**Project Title:** A mixed-methods evaluation of cross-regional workplace health initiatives including a cluster randomised controlled trial (cRCT) of a behaviour change intervention

**Is this project a:**

University of Birmingham Staff Research project

University of Birmingham Postgraduate Research (PGR) Student project

Other (Please specify below)

Click or tap here to enter text.

**Details of the Principal Investigator or Lead Supervisor (for PGR student projects):**

Title: Dr

First name: Laura

Last name: Kudrna

Position held: Research Fellow

School/Department: Applied Heath

Telephone: 0121 414 4388

Email address: [L.Kudrna@bham.ac.uk](mailto:L.Kudrna@bham.ac.uk)

**Details of any Co-Investigators or Co-Supervisors (for PGR student projects):**

ARC = Applied Research Collaboration (NIHR). WM = West Midlands. NWL = Northwest London. NENC = North East and North Cumbria.

1. Dr Lena Al-Khudairy, Senior Research Fellow, University of Warwick, Medical School, ARC WM, Lena.al-Khudairy@warwick.ac.uk
2. Dr Kelly Ann Schmidtke, Assistant Professor, University of Warwick, Medical School, ARC WM, Kelly.A.Schmidtke@warwick.ac.uk
3. Laura Quinn, Medical Statistician, University of Birmingham, Applied Health, ARC WM, l.quinn@bham.ac.uk
4. Dr Lailah Alidu, Research Associate, University of Birmingham, Applied Health, ARC WM, [l.alidu@bham.ac.uk](mailto:l.alidu@bham.ac.uk)
5. Mr James Yates, Research Associate, University of Birmingham, Applied Health, ARC WM, j.yates@bham.ac.uk
6. Prof. Richard Lilford, Professor of Public Health, University of Birmingham, Applied Health, ARC WM, r.j.lilford@bham.a.cuk
7. Paul Bird, Head of Programmes (Engagement), University of Warwick, and Knowledge Mobilisation Lead at WM Academic Health Sciences Network, ARC WM, p.bird.1@warwick.ac.uk
8. Prof. Kate Jolly, Professor of Public Health and Primary Care, University of Birmingham, ARC WM, c.b.jolly@bham.ac.uk
9. Dr Ila Bharatan, Research Fellow, Warwick Business School, ARC WM, Implementation co-Lead, ila.bharatan@wbs.ac.uk
10. Dr Magdalena Skrybant, Patient and Public Involvement Lead, University of Birmingham, ARC WM, m.t.skrybant@bham.ac.uk
11. Niyah Campbell, Patient and Public Involvement and Engagement Officer, University of Birmingham, ARC WM, n.campbell@bham.ac.uk
12. Prof. Graeme Currie, Professor of Public Management, Warwick Business School, ARC WM, Implementation co-Lead
13. Dr Austen El-Osta, Director of the Self-Care Academic Research Unit (SCARU) & Primary Care Research Manager at the School of Public Health, Imperial College London, ARC NWL
14. Dr Dasha Nicholls, Reader in Child Psychiatry, Imperial College London, Honorary Consultant Child and Adolescent Psychiatrist, NIHR NW London ARC Theme Lead for Multimorbidity and Mental Health
15. Dr Benedict Hayhoe, Clinical Lecturer in Primary Care Imperial College London, NIHR NW London ARC Theme for Multimorbidity and Mental Health
16. Dr Kaveh Asanati, Fellow of the Faculty of Occupational Medicine, Consultant Occupational Physician & Honorary Clinical Senior Lecturer at National Heart and Lung Institute, Imperial College London, ARC NWL
17. Prof. Azeem Majeed, Professor of Primary Care and Public Health, and Head of the Department of Primary Care & Public Health at Imperial College London
18. Dr Mackenzie Fong, Fellow in Prevention, Early Intervention and Behaviour Change, Newcastle University, ARC NENC
19. Dr Heather Brown, Senior Lecturer, Population Health Sciences Institute, Newcastle University, ARC NENC
20. Prof. Amelia Lake, Professor of Public Health Nutrition, Teesside University, and Associate Director of Fuse, The Centre for Translational Research in Public Health, ARC NENC
21. Dr Naomi Burn, Lecturer, School of Health and Life Sciences, Teeside University, ARC NENC
22. Patient and Public Involvement Representative (not yet recruited)
23. Joanne Southan, Wellbeing Partnerships Manager, Warwickshire City Council, Jo Southan josouthan@warwickshire.gov.uk

1. Jenny Duggan, Business Development Advisor, Employment & Wellbeing Service, Coventry City Council, jenny.duggan@coventry.gov.uk
2. Sean Russell, Implementation Director for the Mental Health Commission, West Midlands Combined Authority, sean.russell@wmca.org.uk
3. All eight local authorities in Northwest London and Directors of Public Health (Hammersmith & Fulham, Royal Borough of Kensington & Chelsea, Central London/Westminster, Hillingdon, Brent, Harrow, Ealing) – not yet recruited
4. Christine Mead, Director of Westminster Connects, Westminster City Council, cmead@westminster.gov.uk
5. Linda Jackson, Director Health & Wellbeing Hammersmith & Fulham Council, linda.jackson@lbhf.gov.uk
6. Dr Nicola Lang, Director of Public Health, London Borough of Hammersmith & Fulham, nicola.lang@lbhf.gov.uk
7. Susanne Nichol, Better Health at Work Coordinator, Northern Trades Union Congress (TUC), snichol@tuc.org.uk
8. Scott Lloyd, Advanced Public Health Practitioner/Public Health Associate Lead/Clinical Research Network North East & Cumbria BHAWA oversight group member, scott_lloyd@middlesbrough.gov.uk

Further contact details available upon request from PI.

**Details of the student for PGR student projects:**

Title: Click or tap here to enter text.

First name: Click or tap here to enter text.

Last name: Click or tap here to enter text.

Course of study: Click or tap here to enter text.

Email address: Click or tap here to enter text.

**Project start and end dates:**

Estimated start date of project: 01/09/2021
Estimated end date of project: 01/09/2023

**Funding:**

Sources of funding: National Institute of Health Research Applied Research Collaboration Prevent Consortium

#

# Section 2: Summary of Project

Describe the purpose, background rationale for the proposed project, as well as the hypotheses/research questions to be examined and expected outcomes. This description should be in everyday language that is free from jargon - please explain any technical terms or discipline-specific phrases. Please do not provide extensive academic background material or references.

**Background**

The work environment is an important determinant of health and health inequalities, and workplaces play key roles in preventing ill health. International and national organisations like the World Health Organisation and Public Health England encourage implementing employer-led workplace health initiatives tailored to regional contexts, but rigorous evidence for their effectiveness is limited. This research will generate and synthesise new knowledge about the effectiveness of workplace health initiatives across different England regions – North East and North Cumbria, Northwest London, and the West Midlands.

**Work packages**

There is one work package in each region. The North East and North Cumbria team will conduct mixed methods research about the mechanisms of how workplace health initiatives change health and business outcomes. Northwest London will conduct mixed methods research about how to support individuals’ competencies to manage their health through self-care. In the West Midlands, the work package will involve a cluster randomised trial of a behavioural intervention on goal setting, evaluated with mixed methods research.

In the rest of the application form, all statements apply to all work packages unless noted otherwise.

**Work package 1 – theory of change**

Research question: What are the mechanisms through which workplace health initiatives prevent context-relevant ill health and wellbeing outcomes?

We will conduct qualitative interviews and focus groups to develop a theory of change to understand mechanisms through which multilevel (environmental, organisational, individual) strategies promote context-relevant health, business, and organisational-culture outcomes. Our sources include scientific literature, document review of workplace health initiatives, and interviews/focus groups with various stakeholders, including workplace health experts, practitioners, programme staff, and senior workplace staff.

**Work package 2- self care competencies**

Research question: What are the personal drivers & barriers to the sustained adoption of health-seeking self-care behaviours in the workplace?

We will address this research aim by collecting semi-quant data using eSurvey & contextual data from personal interviews/focus group discussions to develop a competency framework on the sustained and routine adoption of health-seeking self-care behaviours within workplace health initiatives. Again, our sources include scientific literature, document review of workplace health initiatives, and interviews/focus groups with various stakeholders, including workplace health experts, practitioners, programme staff, and senior workplace staff.

**Work package 3 – behavioural intervention on goal setting**

Research question: What is the effectiveness of a mental contrasting intervention delivered through workplaces in motivating staff to change their health behaviour and wellbeing?

This part of the research will involve a cluster randomised controlled trial (RCT) in the West Midlands. The trial will test the effectiveness of an established behavioural intervention – mental contrasting. Key aspects of mental contrasting include articulating goals and how to address barriers to achieving them, which our public contributors identified as important drivers of health behaviours. We will co-produce how the intervention is delivered locally (e.g. through ‘wellbeing champions’ already working within organisations, via an email link, in a group session) and implement it in a controlled study with a group of organisations in Coventry.

The RCT is led by the University of Birmingham. The intervention will be delivered to approximately 30 out of 60 workplaces in Coventry who have already signed up to the workplace wellbeing initiative called ‘Thrive at Work’ (https://www.coventry.gov.uk/thriveatwork). The intervention is called ‘mental contrasting’ and it is well-established (https://woopmylife.org/). We will co-produce the mode of delivery (through ‘wellbeing champions’ working within organisations, via an email link, in a group session) with a Patient and Public Involvement Group. The intervention will be evaluated in quantitative online surveys and focus groups.

Cross A, Sheffield D. Mental contrasting for health behaviour change: a systematic review and meta-analysis of effects and moderator variables. Health Psychol Rev. 2019;13(2):209-225. doi:10.1080/17437199.2019.1594332

Mental contrasting website: https://woopmylife.org/en/home

# Section 3: Conduct and location of Project

## **Conduct of project**

## Please give a description of the research methodology that will be used. If more than one methodology or phase will be involved, please separate these out clearly and refer to them consistently throughout the rest of this form.

Mixed methods qualitative and quantitative research including a cluster randomised controlled trial.

Work package 1

Qualitative data will come from focus groups and interviews. We will also review organisational documents and websites, such as the criteria required to achieve workplace health awards (gold, silver, bronze levels of award), and the portfolios that organisations submit to achieve their awards (<https://www.betterhealthatworkaward.org.uk/about-the-award/levels/>). In our data collection tools document, there are guides for qualitative focus groups (data collection tool WP1A) and qualitative interviews (WP1B)

Work package 2

As in Work Package 1, qualitative data will come from interviews and focus groups. Quantitative data will come from e-survey questionnaires. We will also review organisational documents and websites, such as the criteria required to achieve gold, silver, or bronze workplace health awards). In our data collection tools document, there are guides for the qualitative interviews and focus groups (with a joint guide for both, WP2A), and there are questions for the quantitative e-survey (WP2B).

Work package 3

Quantitative data will come from survey questionnaires. Qualitative data will come from focus groups. In our data collection tools document, there are quantitative survey questions (WP3A) and a guide for the qualitative focus groups (WP3B).

**Geographic location of project**

State the geographic locations where the project and all associated fieldwork will be carried out. If the project will involve travel to areas which may be considered unsafe, either in the UK or overseas, please ensure that the risks of this (or any other non-trivial health and safety risks associated with the research) are addressed by a documented health and safety risk assessment, as described in section 10 of this form.

West Midlands, North East and North Cumbria, and Northwest London

# Section 4: Research Participants and Recruitment

**Does the project involve human participants?**

Note: ‘Participation’ includes both active participation (such as when participants take part in an interview) and cases where participants take part in the study without their knowledge and consent at the time (for example, in crowd behaviour research).

Yes

No

*If you have answered NO please go on to Section 8 of this form. If you have answered YES please complete the rest of this section and then continue on to section 5.*

**Who will the participants be?**Describe the number of participants and important characteristics (such as age, gender, location, affiliation, level of fitness, intellectual ability etc.). Specify any inclusion/exclusion criteria to be used.

Across work packages, we expect to conduct around 80-100 qualitative interviews and 700-800 quantitative interviews with staff employed in organisations that participate in workplace health and wellbeing initiatives, such as accreditation and award schemes. We will deliberately sample workplaces from low income areas across regions, including Coventry. Staff must be 16 years of age or older and willing to provide consent.

Work package 1

There will be 40-50 participants included in the mixed methods research.

Work package 2

There will be an additional 40-50 participants included in the mixed methods research.

Work package 3

For the trial, the sample size is 60 workplaces in Coventry, and the 700-800 quantitative interviews are expected within these workplaces (10-15 per workplace, as in our prior work). As this is a cluster trial and the workplaces will be the clusters, we do not have a specific participant target within the workplace clusters and this will depend on sample size. We expect around 120 focus groups (one with each organisation before and after the intervention).

**How will the participants be recruited?**

Please state clearly how the participants will be identified, approached and recruited. Include any relationship between the investigator(s) and participant(s) (e.g. instructor-student). Please ensure that you attach a copy of any poster(s), advertisement(s) or letter(s) to be used for recruitment.

Please see recruitment wording attached.

Work package 1

Participants will be recruited through our collaboration with Co-Is in local authorities and the Trades Union Congress. Workplaces who have signed up to workplace health and wellbeing initiatives and awards will be invited to participate.

Work package 2

Participants will be recruited through our collaboration with Co-Is, including those in local authorities. Workplaces who have signed up to workplace health and wellbeing initiatives and awards will be invited to participate and we will use our contacts with local organisations, too.

Work package 3

Participants will be recruited through our collaboration with Co-Is in Coventry City Council. There are approximately 60 organisations currently participating in a workplace health and wellbeing award in Coventry and these workplaces will be approached by their regular point of contact, the Business Development Advisor, Employment & Wellbeing Service, Coventry City Council, to see if they would like to participate in the trial.

## Section 5: Consent

**What process will be used to obtain consent?**

*Describe the process that the investigator(s) will be using to obtain valid consent. If consent is not to be obtained explain why. If the participants are under the age of 16 it would usually be necessary to obtain parental consent and the process for this should be described in full, including whether parental consent will be opt-in or opt-out.*

A consent from will be provided to participants prior to their research interview. The consent form may be emailed as an attachment, included online in a survey platform such as Qualtrics, read verbally by a researcher employed at a university, or provided on paper in-person (if circumstances allow).

*Please be aware that if the project involves over 16s who lack capacity to consent, separate approval will be required from the Health Research Authority (HRA) in line with the Mental Capacity Act.*

*Please attach a copy of the Participant Information Sheet (if applicable), the Consent Form (if applicable), the content of any telephone script (if applicable) and any other material that will be used in the consent process.*

*Note: Guidance from Legal Services on wording relating to the Data Protection Act 2018 can be accessed at* [*https://intranet.birmingham.ac.uk/legal-services/What-we-do/Data-Protection/resources.aspx*](https://intranet.birmingham.ac.uk/legal-services/What-we-do/Data-Protection/resources.aspx)*.*

**Use of deception?**

*Will the participants be deceived in any way about the purpose of the study?*

Yes

No

*If yes, please describe the nature and extent of the deception involved. Include how and when the deception will be revealed, and the nature of any explanation/debrief will be provided to the participants after the study has taken place.*

Work package 1

There is no deception involved.

Work package 2

There is no deception involved.

Work package 3

While there is no deception involved, we will debrief participants to more fully explain the statement from the consent form that “Some organisations involved in the research will receive extra help implementing content about health and wellbeing at work. “There is now a debriefing statement which will be disseminated via the workplace health and wellbeing ‘champions’ already working within each organisation, such as via email or a noticeboard (depending on the organisation).

Click or tap here to enter text.

## Section 6: Participant compensation, withdrawal and feedback to participants

**What, if any, feedback will be provided to participants?**

*Explain any feedback/ information that will be provided to the participants after participation in the research (e.g. a more complete description of the purpose of the research, or access to the results of the research).*

All work packages: At the end each interview, focus group, and survey, participants will be asked if they have any questions and the researchers can answer them. A summary of the research may be available online and a link provided to participants if this is possible. Participants are told in the PIS that a summary of the findings in non-technical language will be available at the end of the project upon request via email.

**What arrangements will be in place for participant withdrawal?**

*Describe how the participants will be informed of their right to withdraw from the project, explain any consequences for the participant of withdrawing from the study and indicate what will be done with the participant’s data if they withdraw.*

All work packages: Participants will be informed in the participant information sheet and consent form that there are no consequences to withdrawing from the project prior to 31 May 2023. Participants can email Dr. Laura Kudrna and she will arrange for their data to be deleted (L.Kudrna@bham.ac.uk).

*Please confirm the specific date/timescale to be used as the deadline for participant withdrawal and ensure that this is consistently stated across all participant documentation. This is considered preferable to allowing participants to ‘withdraw at any time’ as presumably there will be a point beyond which it will not be possible to remove their data from the study (e.g. because analysis has started, the findings have been published, etc).*

All work packages: Participants will be allowed to withdraw without any consequences prior to 31 May 2023. After that date, their data may be included in a report and it may not be possible to withdraw. This information is in the PIS and consent form

**What arrangements will be in place for participant compensation?**

*Will participants receive compensation for participation?*

Yes

No

*If yes, please provide further information about the nature and value of any compensation and clarify whether it will be financial or non-financial.*

Click or tap here to enter text.

*If participants choose to withdraw, how will you deal with compensation?*

Click or tap here to enter text.

# Section 7: Confidentiality/anonymity

**Will the identity of the participants be known to the researcher?**

*Will participants be truly anonymous (i.e. their identity will not be known to the researcher)?*

Yes

No

**In what format will data be stored?**

*Will participants’ data be stored in identifiable format, or will it be anonymised or pseudo-anonymised (i.e. an assigned ID code or number will be used instead of the participant’s name and a key will kept allowing the researcher to identify a participant’s data)?*

Data will be pseudo-anonymised.

Password-protected files and folders stored on university servers will contain the consent forms, participant names, and an ID code to link to participants’ data.

Qualitative audio/video files and quantitative datasets will be password-protected and stored with the ID code.

Any documents or additional data collected from workplaces or project collaborators at local authorities/trade union congress (absenteeism, presenteeism) will be stored and shared in password-protected files.

“A password-protected Excel file stored on a University server will contain your name, interview date and time, and ID code. This document will be stored separately from your research data. Data from quantitative online interviews will be exported from Qualtrics survey platforms and stored on secure University servers. Qualitative recordings will initially be stored on the Cloud of the University associated with Zoom or Microsoft Teams. Recordings may also be made in password-protected handheld recording devices. All interview links will be password-protected. The interview transcriptions will be downloaded and saved by ID code into a password-protected Zip folder on secure University storage, saved in a password-protected Word or PDF document. After the transcriptions are downloaded, the recordings and transcriptions will be deleted from the cloud storage. Any session notes will be stored electronically in password-protected Word or PDF files on secure University storage. The transcriptions and session notes may be pseudo-anonymised and shared with the University of Teesside, Newcastle University, or Imperial College London. When data are pseudo-anonymised, it means that your name is removed along with any other information that could identify you, such as where you work or live (<https://ico.org.uk/for-organisations/guide-to-data-protection/guide-to-the-general-data-protection-regulation-gdpr/key-definitions/what-is-personal-data/>). The data are pseudo-anonymised and not anonymised because the ID code of your interview could still link your responses to your name if somebody had the Excel document containing your name and ID code. However, the Excel document with your name and ID code will only be accessible by approved University researchers. None of your responses will be attributable to your name in all publications and reports. At the end of the projects, the document linking your name to your ID code will be deleted (31st December, 2023). Anonymised transcripts will be stored for 10 years according to the University guidelines.”

**Will participants’ data be treated as confidential?**

*Will participants’ data be treated as confidential (i.e. they will not be identified in any outputs from the study and their identity will not be disclosed to any third party)?*

Yes

No

*If you have answered no to the question above, meaning that participants’ data will not be treated as confidential (i.e. their data and/or identities may be revealed in the research outputs or otherwise to third parties), please provide further information and justification for this:*

Click or tap here to enter text.

# Section 8: Storage, access and disposal of data

**How and where will the data (both paper and electronic) be stored, what arrangements will be in place to keep it secure and who will have access to it?**

*Please note that for long-term storage, data should usually be held on a secure University of Birmingham IT system, for example BEAR (see* [*https://intranet.birmingham.ac.uk/it/teams/infrastructure/research/bear/index.aspx*](https://intranet.birmingham.ac.uk/it/teams/infrastructure/research/bear/index.aspx)*).*

All work packages

Qualitative recordings will initially be stored on the Cloud of the University associated with Zoom or Microsoft Teams. Recordings may also be made in a password-protected handheld recording devices (as a backup). All interview links will be password-protected.

Next, the qualitative interviews will be downloaded into a password-protected folders on secure University servers and sent to transcription companies. The transcription companies will send back pseudo-anonymised transcripts back in a password-protected document, such as a Zip Folder, Word, or PDF document. After the transcriptions are received, the recordings and transcriptions will be deleted from the Cloud storage. Any session notes will be stored electronically in password-protected Word or PDF files on University servers.

The pseudo-anonymised transcriptions and session notes may be shared between the University of Birmingham, University of Warwick, Imperial College London, Newcastle University, and Teesside University via email.

Data from quantitative online interviews will be exported from survey platforms such as Qualtrics and stored on secure University servers.

Any documents or additional data collected from workplaces or project collaborators at local authorities/trade union congress (absenteeism, presenteeism) will be stored and shared in password-protected files on secure University servers.

Pseudo-anonymised transcripts, session notes, and quantitative datasets will be uploaded to long term storage such as BEAR at the end of the project (https://intranet.birmingham.ac.uk/it/teams/infrastructure/research/bear/beardatashare/index.aspx).

**Data retention and disposal**

*The University usually requires data to be held for a minimum of 10 years to allow for verification. Will you retain your data for at least 10 years?*

Yes

No

*If data will be held for less than 10 years, please provide further justification:*

Click or tap here to enter text.

*What arrangements will be in place for the secure disposal of data?*

Any paper notes will be shredded.

Long-term data storage will be in a secure system such as BEAR and deleted after 10 years.

A request will be made to University IT departments and, if necessary, Zoom and Microsoft Team platforms, to ensure all data and backups are fully deleted from University servers and the Cloud at the end of the project. Principle 6.1 if the Microdata Handling Guide of the UK Data Service will be discussed in relation to deleting the data: https://www.ukdataservice.ac.uk/media/604725/cd171-microdatahandling.pdf

# Section 9: Other approvals required

**Are you aware of any other national or local approvals required to carry out this research?**

*E.g. clearance from the Disclosure and Barring Service (DBS), Local Authority approval for work involving Social Care, local ethics/governance approvals if the work will be carried out overseas, or approval from NOMS or HMPPS for work involving police or prisons? If so, please provide further details:*

Yes, collaborating Universities will submit this form for review by their own ethics teams (University of Warwick, Imperial College London, Newcastle University, Teesside University

**For projects involving NHS staff, is approval from the Health Research Authority (HRA) needed in addition to University ethics approval?**

*If your project will involve NHS staff, please go to the HRA decision tool at* [*http://www.hra-decisiontools.org.uk/research/*](http://www.hra-decisiontools.org.uk/research/) *to establish whether the NHS would consider your project to be research, thus requiring HRA approval in addition to University ethics approval. Is HRA approval required?*

Yes

No

*Please include a print out of the HRA decision tool outcome with your application.*

# Section 10: Risks and benefits/significance

**Benefits/significance of the research**

*Outline the potential significance and/or benefits of the research*

The beneficiaries include employees themselves who may experience fewer health problems, workplaces that may benefit from a healthier and more productive workforce, employers who may change what works to support staff health and wellbeing in their organisations, and economic benefits such as reduced sickness absence, presenteeism, and staff turnover. Incorporating and testing theory means that our academic understanding of implementing behaviourally informed interventions in workplace settings will progress.

**Risks of the research**

*Outline any potential risks (including risks to research staff, research participants, other individuals not involved in the research, the environment and/or society and the measures that will be taken to minimise any risks and the procedures to be adopted in the event of mishap.)* ***Please ensure that you include any risks relating to overseas travel and working in overseas locations as part of the study, particularly if the work will involve travel to/working in areas considered unsafe and/or subject to travel warnings from the Foreign and Commonwealth Office (see*** https://www.gov.uk/foreign-travel-advice)***. Please also be aware that the University insurer, UMAL, offers access to RiskMonitor Traveller, a service which provides 24/7/365 security advice for all travellers and you are advised to make use of this service (see*** [***https://umal.co.uk/travel/pre-travel-advice/***](https://umal.co.uk/travel/pre-travel-advice/)***).***

***The outlining of the risks in this section does not circumvent the need to carry out and document a detailed Health and Safety risk assessment where appropriate – see below.***

All work packages:

The main risk to this project is the collection, storage and/or manipulation of personal/sensitive data. To address this risk, we confirm that we will:

- Use an institutionally approved VPN if working from home.
- Analyse and store data on a University servers only, secured using University VPN.
- Under no circumstances will we store personal data locally onto a home device. In other words, if a University laptop is stored at home, it will not be used to store data.
- Any data not retained in long term storage will be deleted at the end of the project in accordance with section 6.1 of the microdata handling guide: https://www.ukdataservice.ac.uk/media/604725/cd171-microdatahandling.pdf
- Antivirus software will be kept up to date.
- University laptops will be protected by secure passphrases.
- University storage is only accessible via personal authentication with passphrase.
- No data will be stored on portable media.
- The PI has passed the University’s information security and data protection trainings within last year.
- Qualitative interviews will be conducted with headphones so that others cannot hear.
- Participants will be assigned ID numbers so no identifiable information will be published.
- Participant names will not be published in the report alongside quotes. Any identifiable information will be removed from quotes.
- Names or identifiable characteristics will not be used in interim reports or publicly available reports or publications.
- If names or identifiable characteristics are used by participants in qualitative interviews, these will be changed within the transcript to avoid identifying individuals.
- Participants will be fully informed about how their data will be collected, shared, and used in the Participant Information Sheet.
- Although interviewees would have received a Participant Information Sheet along with a copy of the consent form, information will be repeated at commencement of the interview.
- Written consent will be checked prior to arranging interviews.
- Interviewees’ understanding of the research and their rights will be checked at the beginning of the interview by asking a number of closed questions that require a yes or no response, e.g. Do you understand that you can stop this interview at any time?
- Participants will be informed about their right to withdraw from the study at any time before the interviews commence.
- Participants will be made aware that they can stop the interview if they wish to withdraw from the study at any time.
- If participants decide to withdraw their data post interview, every effort will be made to remove their data, however this may not always be possible if publication has already taken place. They will be informed that they will not have to give a reason for withdrawing and this will not affect their circumstances in any way.
- If choosing to withdraw from the study, any data collected before withdrawal will be deleted and quotes will be removed from any written reports if it is possible to do so – as above, if publication has already taken place, this will not be possible (but this will be made clear to participants as part of the consent process in the participant information sheet).

It is also possible that some participants may experience negative emotions from being asked about their health and wellbeing. A signposting document will be available for participants who report any issues and it will include the details of the ethics committee and the principal researcher should in case they want to report an incident.

**University Health & Safety (H&S) risk assessment**

*For projects of more than minimal H&S risk it is essential that a H&S risk assessment is carried out and signed off in accordance with the process in place within your School/College and you must provide a copy of this with your application. The risk may be non-trivial because of travel to, or working in, a potentially unsafe location, or because of the nature of research that will carried out there. It could also involve (irrespective of location) H&S risks to research participants, or other individuals not involved directly in the research. Further information about the risk assessment process for research can be found at* [*https://intranet.birmingham.ac.uk/hr/wellbeing/worksafe/policy/Research-Risk-Assessment-and-Mitigation-Plans-RAMPs.aspx*](https://intranet.birmingham.ac.uk/hr/wellbeing/worksafe/policy/Research-Risk-Assessment-and-Mitigation-Plans-RAMPs.aspx)*.*

Please note that travel to (or through) ‘FCO Red zones’ requires approval by the University’s Research Travel Approval Panel, and will only be approved in exceptional circumstances where sufficient mitigation of risk can be demonstrated.

# Section 11: Any other issues

**Does the research raise any ethical issues not dealt with elsewhere in this form?**

*If yes, please provide further information:*

Click or tap here to enter text.

**Do you wish to provide any other information about this research not already provided, or to seek the opinion of the Ethics Committee on any particular issue?**

*If yes, please provide further information:*

If it is feasible to conduct any in-person research we will revise and resubmit an amendment to the ethics form. Currently, all planned research is virtual.

The signposting document for any participants who report they are struggling with their mental health is attached.

# Section 12: Peer review

**Has your project received scientific peer review?**

Yes

No

*If yes, please provide further details about the source of the review (e.g. independent peer review as part of the funding process or peer review from supervisors for PGR student projects):*

Click or tap here to enter text.

# Section 13: Nominate an expert reviewer

*For certain types of project, including those of an interventional nature or those involving significant risks, it may be helpful (and you may be asked) to nominate an expert reviewer for your project. If you anticipate that this may apply to your work and you would like to nominate an expert reviewer at this stage, please provide details below.*

Title: Click or tap here to enter text.

First name: Click or tap here to enter text.

Last name: Click or tap here to enter text.

Email address: Click or tap here to enter text.

Phone number: Click or tap here to enter text.

*Brief explanation of reasons for nominating and/or nominee’s suitability:*

Click or tap here to enter text.

# Section 14: Document checklist

*Please check that the following documents, where applicable, are attached to your application:*

Recruitment advertisement

Participant information sheet

Consent form

Questionnaire

Interview/focus group topic guide

*Please proof-read study documentation and ensure that it is appropriate for the intended audience before submission.*

# Section 15: Applicant declaration

*Please read the statements below and tick the boxes to indicate your agreement:*

I submit this application on the basis that the information it contains is confidential and will be used by the University of Birmingham for the purposes of ethical review and monitoring of the research project described herein, and to satisfy reporting requirements to regulatory bodies. The information will not be used for any other purpose without my prior consent.

The information in this form together with any accompanying information is complete and correct to the best of my knowledge and belief and I take full responsibility for it.

I undertake to abide by University Code of Practice for Research (https://www.birmingham.ac.uk/Documents/university/legal/research.pdf) alongside any other relevant professional bodies’ codes of conduct and/or ethical guidelines.

I will report any changes affecting the ethical aspects of the project to the University of Birmingham Research Ethics Officer.

I will report any adverse or unforeseen events which occur to the relevant Ethics Committee via the University of Birmingham Research Ethics Officer.

**Please now save your completed form and email a copy to the Research Ethics Officer, at aer-ethics@contacts.bham.ac.uk. As noted above, please do not submit a paper copy.**
