# Supplementary material for: Protocol for a cluster randomised waitlist-controlled trial of a goal-based behaviour change intervention for employees in workplaces enrolled in health and wellbeing initiatives
Source: PLoS One. 2023 Sep 28;18(9):e0282848. doi: 10.1371/journal.pone.0282848 (PMC10538707; doi:10.1371/journal.pone.0282848)
Supplement: S12 File — (ZIP) [file pone.0282848.s012.zip › consent_v5.docx]

# All work packages

# Workplace Health and Wellbeing - Consent Form

# Project title: A mixed-methods evaluation of cross-regional workplace health initiatives including a cluster randomised controlled trial (cRCT) of a behaviour change intervention

This information is being collected as part of a research project concerned with workplace health and wellbeing by a collaboration of researchers from the University of Birmingham, University of Warwick, Imperial College London, Newcastle University, and Teesside University.

The information which you supply and that which may be collected as part of the research project will be entered into a filing system or database and will only be accessed by authorised personnel involved in the project. The information will be retained by the University and will only be used for the purpose of research, and statistical and audit purposes. By supplying this information you are consenting to the University storing your information for the purposes stated above.

The information will be processed by the University in accordance with the provisions of the GDPR and Data Protection Act 2018. No identifiable personal data will be published.

I consent to take part in:  for yes  for no (select all that apply)

The online/paper survey (questions in a web browser in a link that is emailed to you or on paper)

The online/face to face interview (one-on-one discussion with a researcher over Zoom or Microsoft Teams or in person)

The online/face to face discussion (group discussion over Zoom or Microsoft Teams or in person)

Where applicable, I consent to:  for yes  for no (select all that apply)

my interview/focus group being audio recorded

my interview/focus group being video recorded

I confirm that:  for yes  for no

I have read and understand the participant information leaflet for this study*

I have had the opportunity to ask questions and receive satisfactory answers*

I understand that my participation is voluntary and that I am free to withdraw at any time without giving any reason*

I understand that if I withdraw my data before 31 May 2023 it will be removed from the study and will be destroyed*

I understand that my personal data will be processed for the purposes detailed above, in accordance with the GDPR and Data Protection Act 2018*

I understand that my pseudo-anonymous data may be looked at by staff from the University of Birmingham, University of Warwick, Imperial College London, Newcastle University, and Teesside University*

I understand that interview/focus group recordings may be sent to an external company for transcription*

Where applicable, I understand that my quotes may be used in reports and publications but these will not include any identifiable characteristics (quotes will be anonymised)

Based upon the above, I agree to take part in this study*

*Required

Name of participant…………………………. Date……………………… Signature…………………………..
