# Supplementary material for: Protocol for a cluster randomised waitlist-controlled trial of a goal-based behaviour change intervention for employees in workplaces enrolled in health and wellbeing initiatives
Source: PLoS One. 2023 Sep 28;18(9):e0282848. doi: 10.1371/journal.pone.0282848 (PMC10538707; doi:10.1371/journal.pone.0282848)
Supplement: S12 File — (ZIP) [file pone.0282848.s012.zip › debriefing wording.docx]

# Work package 3 only

**Workplace Health and Wellbeing - Participant Information Sheet – Further Information**

# Project title: A mixed-methods evaluation of cross-regional workplace health initiatives including a cluster randomised controlled trial (cRCT) of a behaviour change intervention

**Investigator (s): University of Birmingham, University of Warwick, Imperial College London, Newcastle University, Teesside University**

Recently you may have participated in an activity about your health and wellbeing for ‘Thrive at Work’. In the activity, people were asked to set a goal and think about how they could go about achieving and accomplishing the goal. As part of the activity, researchers were testing whether setting goals in this way improves the chances of success and wellbeing. Thank you for taking part.

Would you like further information? Email the Project Investigator Dr Laura Kudrna, Research Fellow, University of Birmingham, L.Kudrna@bham.ac.uk
