# Supplementary material for: Protocol for a cluster randomised waitlist-controlled trial of a goal-based behaviour change intervention for employees in workplaces enrolled in health and wellbeing initiatives
Source: PLoS One. 2023 Sep 28;18(9):e0282848. doi: 10.1371/journal.pone.0282848 (PMC10538707; doi:10.1371/journal.pone.0282848)
Supplement: S12 File — (ZIP) [file pone.0282848.s012.zip › pis_v5.docx]

# All work packages

**Workplace Health and Wellbeing - Participant Information Sheet**

# Project title: A study of a goal-setting intervention for employees in workplaces on the Thrive at Work programme

# A mixed-methods evaluation of cross-regional workplace health initiatives including a cluster randomised controlled trial (cRCT) of a behaviour change intervention

**Investigator (s): University of Birmingham, University of Warwick, Imperial College London, Newcastle University, Teesside University**

You are invited to take part in a research study. Before you decide, you need to understand why the study is being done and what it would involve for you. Please take the time to read the following information carefully. Please contact Dr Laura Kudrna at the University of Birmingham (L.Kudrna@bham.ac.uk) if there is anything that is not clear or if you would like more information.

**What is this project about?**

This project is about workplace health and wellbeing. We want to find out if there are ways to improve people’s health and wellbeing by better understanding what happens to them at work. We are talking to people and analysing questions from surveys. To evaluate the effectiveness of efforts to improve workplace health and wellbeing, we would like to talk to employees of organisations like yours that have workplace health and wellbeing initiatives.

**Do I have to take part?**

No, you do not have to take part. You will not be penalised if you do not take part. It is entirely up to you to decide.

**What will happen to me if I take part?**

A researcher will contact you to arrange a research activity. The researcher will be from the University of Birmingham, University of Warwick, Imperial College London, Newcastle University, or Teesside University. You could be asked to take part in:

- **An ‘online/paper survey’**, where you would be asked to click on a link that is emailed to you and answer around 20 minutes of survey questions online or on paper in person.
- **An ‘online/face to face interview’**, where you would be invited to have a one-on-one interview with a researcher over Zoom or Microsoft Teams, which should last around for around 45 minutes.
- **An ‘online/ face to face discussion’**, you would be invited to have an interview with a researcher and/ or other participants in a ‘focus group’ over Zoom or Microsoft Teams or in person, which should last around for around 45 minutes.

You will be asked some questions about who you are, where you work and your experience with health and wellbeing at work. Some of the organisations involved in the research will receive extra help implementing content about health and wellbeing at work.

If you choose to participate, we will ask you to sign a consent form to confirm that you have agreed to take part. You will be free to withdraw at any time, without giving a reason, and this will not affect you or your circumstances in any way. If choosing to withdraw from the study, any data collected before withdrawal will be deleted and quotes will be removed from any written reports if it is possible to do so. However, after 31 May 2023, this may not be possible because publication may have already taken place.

We ask that participants in the online discussion keep each other’s identities and contributions confidential outside the group.

**Who is organising and funding the study?**

The study is funded by National Institute for Health Research (NIHR) Applied Research Collaborations in the West Midlands, Northwest London, North East and North Cumbria, and the NIHR Prevent Consortium. More information about the funding is available online here: <https://arc-w.nihr.ac.uk/news/nihr-arcs-national-research-priorities/>. There are researchers from the University of Birmingham, University of Warwick, Imperial College London, Newcastle University, Teesside University organising the project. The project has received ethics approval from the University of Birmingham’s Science, Technology, Engineering and Mathematics Research Ethics Committee.

**Why was I asked to take part in the project?**

You were asked to take part in the study because people who work at your organisation have said they are interested in workplace health and wellbeing. We would like to understand your experience of health and wellbeing at work and ask you some questions about it.

**What are the dates for the interviews?**

Interviews will take place between September 2021 and September 2023.

**How long will the interview last?**

The online surveys are expected to last around 20 minutes and the online discussions are expected to last around 45 minutes (depending on your answers).

**Will my personal information be kept private?**

Yes, all information about you will be kept very safe and private. Your name will not be used in any reports so no one will know what you have said. If you say anything that makes us think that you or anyone else may be at risk of harm, then we may have to speak to someone outside of the research team. Your interviews may be transcribed by computer software on Zoom or Microsoft Teams or a professional transcription company. These transcriptions will be password-protected. Direct quotes may be used in reports but this will not include any identifiable characteristics (quotes will be anonymised).

If you report anything to us that suggests you are at risk of harming yourself or others we may need to report our safeguarding concerns to the Principal Investigator (Laura Kudrna) at the University of Birmingham, Austen El-Osta at Imperial College London or Amelia Lake at Teesside University.

**What will happen to my data?**

“A password-protected Excel file stored on a University server will contain your name, interview date and time, and ID code. This document will be stored separately from your research data. Data from quantitative online interviews will be exported from Qualtrics survey platforms and stored on secure University servers. Qualitative recordings will initially be stored on the Cloud of the University associated with Zoom or Microsoft Teams. Recordings may also be made in password-protected handheld recording devices. All interview links will be password-protected. The interview transcriptions will be downloaded and saved by ID code into a password-protected Zip folder on secure University storage, saved in a password-protected Word or PDF document. After the transcriptions are downloaded, the recordings and transcriptions will be deleted from the cloud storage. Any session notes will be stored electronically in password-protected Word or PDF files on secure University storage. The transcriptions and session notes may be pseudo-anonymised and shared with the University of Teesside, Newcastle University, or Imperial College London. When data are pseudo-anonymised, it means that your name is removed along with any other information that could identify you, such as where you work or live (<https://ico.org.uk/for-organisations/guide-to-data-protection/guide-to-the-general-data-protection-regulation-gdpr/key-definitions/what-is-personal-data/>). The data are pseudo-anonymised and not anonymised because the ID code of your interview could still link your responses to your name if somebody had the Excel document containing your name and ID code. However, the Excel document with your name and ID code will only be accessible by approved University researchers. None of your responses will be attributable to your name in all publications and reports. At the end of the projects, the document linking your name to your ID code will be deleted (31 December 2023). Your anonymised transcripts will be stored for 10 years according to the University guidelines.”

**What are the benefits and risks associated with taking part in this research?**

Sometimes people benefit from taking part in research like this because they pay more attention to their own health and wellbeing as a result, which can improve it. It is an opportunity to contribute to improving our understanding of health and wellbeing, which may benefit you and others if this knowledge is successfully applied later on to improve health and wellbeing. There are minimal risks involved with your participation in this research, although we will ask for some of your time that you could spend doing other things.

If you are experiencing mistreatment at work, you can contact your Human Resources department or the independent Advisory, Conciliation and Arbitration Service (ACAS) (<https://www.acas.org.uk/> or 0300 123 1100).

**Can I find out the results of the research?**

Yes. After we finish collecting and analysing the data we will write publications and reports about what we have found and share them so that other people can use them to better understand and improve individual and community wellbeing. A summary of the findings in non-technical language will be available at the end of the project and is available upon request from Dr Laura Kudrna (L.Kudrna@bham.ac.uk.).

**Who can I contact if I have questions?**

If you have any questions or concerns regarding the research then please do not hesitate to contact Dr Laura Kudrna, Research Fellow, University of Birmingham, [L.Kudrna@bham.ac.uk](mailto:L.Kudrna@bham.ac.uk) or Charlotte Rothwell, Senior Research Associate, Newcastle University, [charlotte.rothwell@newcastle.ac.uk](mailto:charlotte.rothwell@newcastle.ac.uk) or Prof Amelia Lake, Professor of Public Health Nutrition, Teesside University, [Amelia.Lake@Tees.ac.uk.](mailto:Amelia.Lake@Tees.ac.uk) If you have any concerns/complains about the research process you can also contact the University of Ethics Committee aer-ethics@contacts.bham.ac.uk.
