# Supplementary material for: Protocol for a cluster randomised waitlist-controlled trial of a goal-based behaviour change intervention for employees in workplaces enrolled in health and wellbeing initiatives
Source: PLoS One. 2023 Sep 28;18(9):e0282848. doi: 10.1371/journal.pone.0282848 (PMC10538707; doi:10.1371/journal.pone.0282848)
Supplement: S12 File — (ZIP) [file pone.0282848.s012.zip › recruitment wording_v3.docx]

**Applies to All Work Packages**

Dear [name],

I hope this email finds you well.

I am contacting you because your workplace is part of [fill in as appropriate – [Thrive at Work](https://www.coventry.gov.uk/info/134/thrive_at_work/3105/thrive_at_work_workplace_wellbeing_overview), [Better Health at Work](https://www.betterhealthatworkaward.org.uk/), etc). We would like to ask you some questions about your experience with health and wellbeing at work [(WP3 only) invite you to set a new goal about your health and wellbeing, as appropriate – to be confirmed with co-production patient and public involvement activity].

We would very much like to include you in our research.

Would it be possible to speak with you at your convenience at some point in the next few weeks? The virtual discussion would last around 45 minutes, depending on your answers / Here is a link to participate in the research, which should take around 20 minutes to complete. [note consent form sent before interview or embedded within study link]

Thank you. I look forward to hearing from you.

Sincerely,

Name of researcher

Signature of researcher
