# Supplementary material for: Protocol for a cluster randomised waitlist-controlled trial of a goal-based behaviour change intervention for employees in workplaces enrolled in health and wellbeing initiatives
Source: PLoS One. 2023 Sep 28;18(9):e0282848. doi: 10.1371/journal.pone.0282848 (PMC10538707; doi:10.1371/journal.pone.0282848)
Supplement: S12 File — (ZIP) [file pone.0282848.s012.zip › signposting_v3.docx]

**Applies to all work packages**

**Accessing support for your health and wellbeing**

If you have concerns about your mental or physical health please speak with your GP. You can also visit NHS Choices via [http://www.nhs.uk/](http://www.nhs.uk/pages/home.aspx). If it is an emergency you should call 999.

If you are experiencing mistreatment at work, you can contact your Human Resources department or the independent Advisory, Conciliation and Arbitration Service (ACAS) (<https://www.acas.org.uk/> or 0300 123 1100).

Additional resources for mental health can be found here:

Psychiatric intervention is available via referral from your GP. In a mental health emergency, crisis intervention teams are available where Approved Social Workers (ASWs) can attend to discuss the situation on site. There are also walk-in centres at local hospitals for emergency admissions. A GP or other clinical practitioner can recommend a hospital admission as a helpful respite.

*SANEline*: 12noon-2am daily 0845 7678000. SANEline is a national out-of-hours mental health helpline offering specialist emotional support, guidance and information to anyone affected by mental illness, including family, friends and carers (http://www.sane.org.uk/what_we_do/support/).

*Samaritans* 0845 909090 or call 116 123 or email [jo@samaritans.org](mailto:jo@samaritans.org). Samaritans offer a safe place for you to talk any time you like, in your own way – about whatever’s getting to you (https://www.samaritans.org/).

*Mind* call 03001233393 or text 86463 or email [info@mind.org.uk or](mailto:info@mind.org.uk%20or) visit [www.mind.org.uk/information](http://www.mind.org.uk/information). Mind provides [advice and support](https://www.mind.org.uk/information-support/) to empower anyone experiencing a mental health problem. Mind [campaign](https://www.mind.org.uk/news-campaigns/campaigns/) to improve services, raise awareness and promote understanding.

*Royal College of psychiatrists* call 020 7235 2351 or 020 7977 6655 or email [reception@rcpsych.ac.uk](mailto:reception@rcpsych.ac.uk) or visit [www.rcpsych.ac.uk/info](http://www.rcpsych.ac.uk/info). TheRoyal College is the professional and educational body for psychiatrists in the United Kingdom. They:

- set standards and promote excellence in psychiatry and mental healthcare
- lead, represent and support psychiatrists
- work with patients, carers and their organisations.

*Rethink mental illness* call 0300 5000 927 or visit [www.rethink.org](http://www.rethink.org/). Rethink help millions of people affected by mental illness by challenging attitudes, changing lives. They provide expert, accredited advice and information to everyone affected by mental health problems.

*Depression and mental health resources* please visit [www.psyweb.com](http://www.psyweb.com/).

[*Papyrus*](https://papyrus-uk.org/hopelineuk/) *Prevention of young suicide* free on 0800 068 41 41 or text 07786 209697 or visit <https://papyrus-uk.org/> **if you're under 35**. PAPYRUS is the national charity dedicated to the prevention of young suicide.

If you have any concerns/complains about the research process you can contact the Ethics Committee - aer-ethics@contacts.bham.ac.uk.
