# Supplementary material for: Protocol for a cluster randomised waitlist-controlled trial of a goal-based behaviour change intervention for employees in workplaces enrolled in health and wellbeing initiatives
Source: PLoS One. 2023 Sep 28;18(9):e0282848. doi: 10.1371/journal.pone.0282848 (PMC10538707; doi:10.1371/journal.pone.0282848)
Supplement: S12 File — (ZIP) [file ethical approval letter for wp1 wp2 jan 2022.pdf › ethical approval letter for wp1 wp2 jan 2022.pdf]

Application for Ethical Review ERN_21-0744

Samantha Waldron (Research Support Services)

Wed 12/01/2022 08:49

To: Laura Kudrna (Applied Health Research) <L.Kudrna@bham.ac.uk>;

Dear Dr Kudrna

Re: "A mixed-methods evaluation of cross-regional workplace health initiatives including a cluster randomised controlled trial (cRCT) of a behaviour change intervention"
Application for Ethical Review ERN_21-0744

Thank you for your application for ethical review for the above project, which was reviewed by the Science, Technology, Engineering and Mathematics Ethical Review Committee.

On behalf of the Committee, I confirm that WP1 and WP2 now have full ethical approval.

I would like to remind you that any substantive changes to the nature of the study as described in the Application for Ethical Review, and/or any adverse events occurring during the study should be promptly brought to the Committee's attention by the Principal Investigator and may necessitate further ethical review.

Please also ensure that the relevant requirements within the University's Code of Practice for Research and the information and guidance provided on the University's ethics webpages (available at https://intranet.birmingham.ac.uk/finance/accounting/Research-Support-Group/Research-Ethics/Links-and-Resources.aspx) are adhered to and referred to in any future applications for ethical review. It is now a requirement on the revised application form (https://intranet.birmingham.ac.uk/finance/accounting/Research-Support-Group/Research-Ethics/Ethical-Review-Forms.aspx) to confirm that this guidance has been consulted and is understood, and that it has been taken into account when completing your application for ethical review.

Please be aware that whilst Health and Safety (H&S) issues may be considered during the ethical review process, you are still required to follow the University's guidance on H&S and to ensure that H&S risk assessments have been carried out as appropriate. For further information about this, please contact your School H&S representative or the University's H&S Unit at healthandsafety@contacts.bham.ac.uk.

Kind regards,

Ms Sam Waldron (she/her)
Research Ethics Officer
Research Support Group
University of Birmingham
Email: S.M.Waldron@bham.ac.uk
Video/phone: If you would like to arrange a Teams/Zoom/telephone call, please email me and I will get in touch with you as soon as possible.
Postal address**:** Ms Sam Waldron, Finance Office, University of Birmingham, c\o Room 106 Aston Webb, B Block, Edgbaston, Birmingham, B15 2TT.

Web: https://intranet.birmingham.ac.uk/finance/RSS/Research-Support-Group/Research-Ethics/index.aspx
Click Research Governance for further details regarding the University's Research Governance and Clinical Trials Insurance processes, or email researchgovernance@contacts.bham.ac.uk with any queries relating to research governance.

Notice of Confidentiality:
The contents of this email may be privileged and are confidential. It may not be disclosed to or used by anyone other than the addressee, nor copied in any way. If received in error please notify the sender and then delete it from your system. Should you communicate with me by email, you consent to the University of

Birmingham monitoring and reading any such correspondence.
