# Supplementary material for: Protocol for a cluster randomised waitlist-controlled trial of a goal-based behaviour change intervention for employees in workplaces enrolled in health and wellbeing initiatives
Source: PLoS One. 2023 Sep 28;18(9):e0282848. doi: 10.1371/journal.pone.0282848 (PMC10538707; doi:10.1371/journal.pone.0282848)
Supplement: Attachment — Submitted filename: Response to reviewers.docx [file pone.0282848.s013.docx]

**Reviewer #1: This manuscript describes a proposed study design and protocol aiming to evaluate a mental contrasting intervention for achievement of health and well-being goals in the workplace.

In contrast to the stated rationale of showing an effect on health behaviour (p. 3-4), the objectives of the study (p.5) are all concerned with the participants’ perception of health and health-related behaviour. Indeed, the main outcomes are participants’ self-reported evaluation of their progress with respect to the goals they set at baseline. Several factors may be expected to influence these perceptions, including the expectations which the participants associate with the intervention, to which they are not blinded. Therefore, the results will not allow direct conclusions concerning the actual, objective effects on health and health behaviour, which will not be determined.

While these limitations apply to many studies especially of psychological interventions, the very subjective nature of the outcomes and the fact that participants are fully aware of the aims of the study and of their allocated intervention make them especially acute in this proposed study. They might be mitigated by veiling the study aim for the participants prior to the intervention, as seems to have been done by Oettingen, Mayer and Brinkmann (Ref. 18), and/or by asking the participants to assess their current health status and behaviour rather than related to baseline. The use of a concomitant ‘placebo’ intervention in the wait-list group might help to reduce disparity in expectations in the two groups. Failing this, these limitations should be described more fully and the planned procedure and questionnaire should be justified in detail.**

*Thank you for raising the important points about the use of self-reported health rather than objective health, expectations participants associate with the intervention, and mitigation options. While we would like to use measures of objective health, we are limited by practical constraints (access to the workplaces) and resource constraints (funding availability). We have expanded where we discuss the limitations of self-reported health: “Finally, all outcomes are self-reported. This is a limitation because no conclusions can be drawn about objective effects on health or health behaviour, and there could be response bias.”*

*While we would like to fully veil the study aim as by Oettingen, Mayer and Brinkmann (Ref. 18), our experience with employers was that they wanted to know the type of content within the sessions before committing to them. We do ask that employers not share the description of the sessions with staff, rather describing them more generally as ‘goal setting sessions’ or ‘going from ‘want’ to ‘do’’. In our questionnaire, we ask if participants have heard of WOOP or mental contrasting plus implementation intentions before (S8 – Q20). Thus, we will be able to assess if the study aim was sufficiently protected.*

*Following the advice of Reviewer 2, our new approach to a concomitant ‘placebo’ intervention in the wait-list group is to focus on the ‘W’ of WOOP and go through its key components (see new S3). Thus we are comparing the ‘W’ of WOOP (received by both intervention and control in the first session) versus the ‘OOP’ (received only by the intervention group in the first session). Participants will not be told if they are in the intervention group, which is now clarified under the section Blinding (masking).*

**It is important that the choices of self-set goal at baseline are broadly comparable between the intervention and waitlist groups. If possible, participants should remain blind to allocation until these goals have been set. At data analysis, choices should be presented descriptively (e.g. by building categories) and compared between groups.**

*We agree this is important. The researcher will not be aware of the group allocation until the goals are set – thus, neither will the participants, and the participants are never told if they are in the intervention or control group. We now include descriptive choices for goals (see S8, Q25: What was your wish about? Health, social relationships, work performance, academic, other).*
 **Minor comments:**

**1. Please explain why a Bayesian (linear mixed) model is planned, since there does not seem to be a concretely relevant prior for the effect.**

*Thank you for your comment. The use of Bayesian analysis using an uninformative priors allows us to provide posterior probabilities for the effect of the intervention instead of dichotomising the results based on p-values.*

**2. Multiple imputation (p.15) is proposed for dealing with missing covariates in a secondary analysis. It would be good to give a reference for this technique.**

*Thank you for your comment. A reference for the multiple imputation methods has been added:* Carpenter J, Kenward M. Multiple imputation and its application. John Wiley & Sons; 2012 Dec 21.

**3. A statistician is supposed to check data for accuracy (p.16) – is this possible, or do you mean plausibility?**

*Thank you for your comment. Yes you are correct and the text had been updated to reflect this.*

**Reviewer #2: Review of Registered Report Protocol PONE-D-22-11119 submitted to PLOS ONE entitled: “A cluster randomised wait-list controlled trial of a goal-setting behavior change intervention for employees in workplaces enrolled in workplace health and well-being initiatives”.

The present protocol describes an intervention study geared at supporting behavior change in the workplace context. The intervention employs a behavior change strategy called WOOP (Wish, Outcome, Obstacle, Plan) which in the scientific literature stands for mental contrasting (MC or WOO) by itself or combined with implementation intentions (MCII or WOOP). Mental contrasting with or without implementation intentions is well tested in the health domain but hardly in the work domain. In fact, the proposal argues that the innovation of the proposed study is that only three studies focus on mental contrasting or WOOP in the workplace and has never been systematically examined in a broad sample of workers during their everyday lives. Impressively, the authors want to address 60 organizations with 30 organizations in the intervention group and 30 organizations in the waitlisted control group. The wishes are supposed to tackle desired futures from the health and well-being domains.

The protocol includes a host of details that is relevant for administrative purposes, is well written, and gives an overview about what the authors are planning to do. The research question asking for the acceptance and effects of WOOP in the workplace is very important, and because the protocol aims at a relatively large sample of organizations and participants, the study is well worth to be conducted. The materials are in the most part well described.

Unfortunately, I am a bit unclear about my role as a reviewer. Judging from the timetables, the study has already started. The question then is, how much can the authors still change in the protocols? I think the study should be done, especially after all the effort of obtaining the contacts to the organizations. However, they are still a host of details which should be improved when it comes to design and instructions of the intervention. In the following, I provide examples on how the materials could be refined according to the theoretical and empirical principles of WOOP. I hope refinement is still possible before the beginning of the study.**

*Thank you for the helpful suggestions to refine, which we were able to incorporate and pilot since receiving your comments. We detail our revisions below.*

**I might not fully understand in what order and in what time frame the instructions are given. Do the authors assume that they can have the baseline filled out and the WOOP exercise taught in 45 minutes to an hour? Have the authors piloted that time frame? I'm also unclear how many participants should be in one group, how the researchers are trained to convey WOOP, how often WOOP is practiced in the first instruction session, and how the materials (participants take them home?) are explained. Plus, what are the fidelity criteria, etc.**

*In terms of timeframe, yes, we piloted the timeframe for the questionnaire and the WOOP exercise. Thus far we have not exceeded 50 minutes for both.*

*In terms of group size, we expect that there will be 10-30 employees in each group: “The key contacts in organisations will be asked to invite volunteer employees to participate until there are at least 10 and no more than 30 employees per organisation.”*

*In terms of training for the researcher to convey WOOP, we have updated the information on ‘Who’ in S4:*

*Who provided. A member of the research team (MSc+) familiar with literature and use of mental contrasting, implementation intentions, and WOOP interventions will provide the sessions. They will have PowerPoint slides and some text to follow during the session. All members of the research team running the sessions will watch videos and listen to audios from the WOOP website (woopmylife.org), attend a training session run by [name of co-author, blind for peer review], and pilot the sessions – receiving feedback from other researchers.*

*WOOP is practiced once in the first instruction session and the handout materials are provided to participants so that they can take them home. Participants are told they can WOOP every day. To confirm that manipulation of OOP occurred as planned (fidelity), researchers delivering the sessions fill out a reporting form and one of the questions is, “Did you deliver the intervention or control session as indicated in the envelope?” This information is now updated in S4.*

**It would be important that the interventionists strictly adhere to the instructions and that the instructions are better standardized. WOOP is a sensitive animal and small changes in wording might have big effects, as the experimental data show. In that respect, as nice as it is that the authors want to give freedom in delivery to the interventionists and want to leave the setting unspecified (Zoom, in person), there is the danger of diluting the WOOP effects. Experimental control should be guaranteed, at least as much as possible. Certainly, we need a balance between the autonomy of the interventionist and the sensitivity of the WOOP exercise, but it would be helpful to twist the WOOP instructions and examples (especially at the beginning) more in the direction of being in line with the WOOP principles plus towards being standardized. To find a compromise between standardization and autonomy of the interventionist the authors may consult the woopmylife.org website. Here one can also speak to the researchers who generated the strategy.**

*Thank you for the suggestion to better standardise the instructions. Previously, we had followed National Institute for Health Research principles and guidance on adapting interventions for a local context using the advice of public contributors (*[*https://www.nihr.ac.uk/health-and-care-professionals/engagement-and-participation-in-research/involve-patients.htm*](https://www.nihr.ac.uk/health-and-care-professionals/engagement-and-participation-in-research/involve-patients.htm)*). However, we appreciate that a balance between the autonomy of the interventionist and the sensitivity of the WOOP exercise are needed.*

*As suggested, we contacted the woopmylife.org website to ask about standardization versus the autonomy of the interventionist. We gratefully received the advice that: “it is crucial to watch out for the standardization of the features WOOP that are responsible for the effects (e.g., four steps, order of the four steps, imagery, specificity, etc.).” In our intervention, the four steps, order of the four steps, use of imagery, and specificity were preserved.*

*After careful deliberation, including discussion with our public involvement liaison and co-authors, we decided to adhere to the material more close to prior WOOP studies that align with material on on woopmylife.org. We could not use the exact website material is it is copyrighted. The studies we used to inform the intervention materials were:*

*Schweiger Gallo I, Bieleke M, Alonso MA, Gollwitzer PM, Oettingen G. Downregulation of anger by mental contrasting with implementation intentions (MCII). Front Psychol. 2018 Oct 4;9:1838.*

*Von Weichs V, Krott NR, Oettingen G. The Self-Regulation of Conformity: Mental Contrasting With Implementation Intentions (MCII). Front Psychol. 2021 Jun 2;12:546178.*

*Gollwitzer PM, Mayer D, Frick C, Oettingen G. Promoting the self-regulation of stress in health care providers: An internet-based intervention. Front Psychol. 2018 Jun 15;9:838.*

*We revised the Powerpoint slides and notes and re-piloted the sessions. One of our considerations was our desire to conduct an independent evaluation to guide scientific results and general conclusions (Lilford, 2018). Independent evaluations add credibility by having those who designed the intervention separate from those who evaluated it – reducing potential bias. The new sessions are in S3 and some of the changes are described below in response to the suggested changes to the instructions.*

*Unfortunately, it was not possible to standardise the mode of delivery. Some workplaces on the local government programme require virtual sessions because their employees are remote, whereas others required in-person sessions. We find the workplaces are unwilling to participate if they cannot choose.*

*Lilford RJ. Implementation science at the crossroads. BMJ quality & safety. 2018 Apr 1;27(4):331-2.*

**Let me specify some points to change in the instructions (without that I can be exhaustive here):**
 **First the authors talk about a goal setting intervention. WOOP, however, starts with the Wish, not with the goal, and the research on mental contrasting makes a strong point that people generate wishes and concerns about desired futures rather than goals. In fact, only after mental contrasting wishes might turn into committal goals. It is critical that participants understand that they come up with a wish, that they are searching for. For finding their wish, participants need to be calm and slow, need to understand that their wish should be “dear to their heart” and truly important. By simply naming a goal participants will not achieve finding this wish (as we have so many goals on the top of our heads). The first step in WOOP, then, is foraging for a wish. Please consult the woopmylife.org website for relevant instructions. The authors can download the WOOP kit and for the tone and slowness also watch the instructional videos. In the instructions of the present protocol, the introduction of the goal at the beginning and then making the first WOOP regarding that goal might mislead people.

In the same vein, it would be helpful if the authors relinquish the term goal setting. The term goal often implies being overladen with pressure and demands from the outside world. Therefore, if the study still can be changed, it would be helpful to let go from the term goal setting when introducing the instructions. Also, to not confuse people, the term WOOP instead of MCII might be enough. Two terms are an unnecessary load.**

*Thank you for these suggestions.*

*Regarding finding the wish, we agree that we need to ensure more slowness and mimic the tone on woopmylife.org. Thus, all researchers delivering the sessions now watch the videos and listen to the audios on woopmylife.org. We have taken the wish out of the survey in order to slowly guide participants in the session through finding something truly important and dear to them. The main part of the first session that all participants receive is now thinking about and reflecting on their wish, including its key components (Truly important? Possible to achieve? Challenging? Summarised in no more than a few sentences?) We now ensure plenty of time to really find something that is important to participants (see S3a).*

*Regarding the word goal, we initially included ‘goal’ rather than ‘wish’ on the advice of our Patient and Public Involvement contributors. The contributors had stated that people in the sessions might find the word ‘wish’ to be childish. However, we agree that the word goal could have implications related to pressure and demands from the outside world. Thus, we have largely reverted to using the word ‘wish’.*

*On the point about WOOP instead of MCII, we now only use WOOP to reduce cognitive load.*
 **When introducing WOOP, the examples do not comply with the WOOP principles. They should be referring to wish, outcome, obstacle, and behavior to overcome the obstacle. These four steps should be built on each other. And they should be short and concise (later the instructions talk about 3 to 6 words). Again, please check the woomylife.org website.**

*We have removed all examples to avoid biasing participants. Again, our Patient and Public Involvement contributors thought that having real examples could enhance the credibility of the intervention. Upon reflection, the desire to reduce bias outweighed the desire to enhance the credibility of the intervention.*

**Later the instructions are more in line with the WOOP principles (p. 56 left side). The authors may want to adjust the examples and instructions, make them consistent across the study, and consistent with what has been depicted on the website.**

*The instructions have been revised to align with previously published WOOP material and be consistent across the study. All examples have been removed. To avoid biasing in line with these examples.*
 **But even in the detailed WOOP instructions, the authors might check again: For example, on p. 51 in the instructions the step to find and specify the behavior to overcome the obstacle is missing. This is a crucial step and should be integrated. Or, in the booklet, it is unclear what people need to do, how often, when, etc. Why does the wish have so many lines? P. 56, too many if-then plans might hurt.**

*The booklet has been removed. Instead, participants are given Wish and OOP handouts. The step to find and specify the behaviour to overcome the obstacle is now included.*

**The preparation for the WOOP exercise is missing. To do a WOOP exercise, people need to be slow and calm, and the instructions before the exercise need to explicitly say that. Otherwise, participants will not generate a meaningful wish and will not be able to come up with the best outcome, and internal critical obstacle, nor will they find a behavior to overcome the obstacle. Importantly, they will not be able to vividly imagine the outcome and the obstacle either.**

*Thank you for pointing out this important issue. We agree and now create space for participants to be slow and calm before their wish, see S3a, p5.*

**It is crucial that people understand that the obstacle needs to be an inner obstacle, an obstacle such as an emotion, belief, or habit – not an external obstacle and thus not an excuse. For example, it is not the boss standing in the way, it is my resentment towards my boss which is the problem. Then I can act. If it is the boss themselves, I will feel helpless as I cannot change my boss. The internality of the obstacle needs to be stressed across the instructions.**

*We agree and now use language around inner obstacle such as emotions, beliefs, or habits, see S3b, pages 6-7, S3e, pages 7-9.*

**It would be important, after participants have done the WOOP exercise, to stress that WOOP is an exercise that can be used for life-changing wishes but also in daily life to structure the upcoming day, the upcoming meeting, the long-postponed phone call, the upcoming evening. It is also important to stress that WOOP can and should be used every day, possibly at the same time, for all sorts of wishes and concerns. It is a skill that needs to be practiced. This guidance for the usage of WOOP should be included in the instructions.**

We agree and now use similar language without infringing on the woopmylife.org copyright (we did ask to use the material more precisely but received no reply):

*“You can use WOOP as much or as little ask you like – for small wishes and big wishes, short term wishes, or long term wishes. You could use WOOP when you feel like you have too much on your plate or when there is something you would really like to do for yourself. Some people say they use WOOP everyday and that it helps them in their everyday life and plans for their future. Do not worry if every wish you have does not happen, though. People who use WOOP say they have seen improvements in their work, family life, and relationships. If you use WOOP often, and practice it, you will get better at it. So really get to know it - it can help you to find out what it is you really want and what is it that keep you from going for what you really want. You can go from 'wanting' - and knowing more about your 'wanting' - to doing.”* 

**It is unclear what the interactions between the interventionist and the participants are in the group context; it is also not described how the members of the group are supposed to communicate. Plus, is WOOP given in writing, or with spoken instructions, or a mixture? Again, the question is how standardized the instructions are, a question that is particularly relevant in case the instruction would vary between the different organizations. Randomization is, as I understand by workplace. If that is the case, will workplace vary with other variables? For example, interventionist, group size, intervention context, setting, etc.? If these confounds exist, it would be even more important to standardize instructional materials.**

*We now have clarified beneath the slides where interaction points occur. These were re-developed after re-piloting. In the S3 material, every point of interaction is now noted as **’Interaction’.*

*To summarise, there are several main points for interaction between interventionist and participants:*

- *In the beginning, they are asked about common intention-behaviour gaps: how many of you have wanted to do something to improve your health and wellbeing but it has not happened in the end? (show of hands) S3a, page 2.*
- *Discussion of the usefulness of WOOP (see S3b, page 13; see S3c, pages 1-3).*
- *In the second sessions, the questions from normalisation process theory are interactive (see S3c, page 2; S3d, pages 2-6).*

*WOOP is provided in writing (W and OOP handouts) and participants are taken through these handouts with spoken instructions. Instructions are the same between organisations, apart from the survey and handouts being delivered virtually in the virtual sessions versus on paper in the in-person sessions.*

*Yes randomisation is by workplace – random block sizes of two and four will be used to maintain balance.*
 **As for the dependent variables, why would the authors not try to record objective measures? It has been shown that objective measures are often a better indicator of behavior change after mental contrasting and WOOP (because when using WOOP automatic processes that people cannot report about are responsible for the behavior change) than subjective measures. Would the authors be able to ask other people observe the participants (within the group?), retrieve some performance (e.g., performance evaluations), and well-being indicators (e.g., sick days)?**

*In our previous work, we asked employers about the feasibility of collecting objective data and found it would be difficult within our time and funding constraints. Organisations in the local government programme tend to keep different types of records about sickness absence and performance – thus, the heterogeneity would make it difficult data to analyse. Employees reporting on each other may raise privacy concerns. We note that “future research could examine the effects on observational measures like absenteeism or productivity”, although this could require organisations standardising their approach in advance. We would like to explore this in our future work.*

**In sum, the instructions around WOOP should be made more consistent and in line with the theory and the literature (as guidance see woopmylife.org). The terminology of goal setting may be misleading, and the examples should be adjusted and standardized. In that respect, examples should be provided sparsely. People tend to emulate them ending up with wishes and obstacles that they do not feel strongly about.**

**If questions about details arise, the authors may turn to woopmylife.org, and initiate contact there.**

*Thank you for your detailed and attentive comments and suggestions. The term goal has been largely removed and the examples are no longer included to avoid bias. We contacted woopmylife.org and included their suggestions. We believe our sessions are much stronger as a result.*
