# Supplementary material for: Protocol for a cluster randomised waitlist-controlled trial of a goal-based behaviour change intervention for employees in workplaces enrolled in health and wellbeing initiatives
Source: PLoS One. 2023 Sep 28;18(9):e0282848. doi: 10.1371/journal.pone.0282848 (PMC10538707; doi:10.1371/journal.pone.0282848)
Supplement: Attachment — Submitted filename: Response to Reviewers1.docx [file pone.0282848.s014.docx]

17 January 2023

**Reviewer #3: The author has incorporated much of the responses of the previous reviewers which has clarified and addressed some of the concerns and comments. However there are still a few places where it could benefit from further clarification, such as:**

**>>The timing of the intervention/control sessions - will they be in worktime or not? It is important in a workplace study to be clear about when and where an intervention is being held - this needs to be standardised across the study**

*We agree and have clarified that the intervention will be “delivered to employees from the same organisation in a group setting during working hours.”*

***>>There is mention of a midline as a data collection timepoint (under statistician will check the data) but there is not a description or reference to this in the study description earlier - it seemed to be only 2 data collection points.***

*Thank you for noticing this typo, we have removed reference to the midline.* **>>It is not clear what the participants will be doing in the 4+ weeks between start and finish - will they be carrying out the woop strategy whenever they wish? I think the study would benefit from some sort of quantifying of participant use of the intervention over the study period.**

*This issue was also raised during our piloting and participants in the intervention group are told they can keep using WOOP (see S3b, page 12):*

*“You can use WOOP as much or as little ask you like – for small wishes and big wishes, short term wishes, or long term wishes. You could use WOOP when you feel like you have too much on your plate or when there is something you would really like to do for yourself. Some people say they use WOOP everyday and that it helps them in their everyday life and plans for their future. Do not worry if every wish you have does not happen, though. People who use WOOP say they have seen improvements in their work, family life, and relationships. If you use WOOP often, and practice it, you will get better at it. So really get to know it - it can  help you to find out what it is you really want and what is it that keep you from going for what you really want. You can go from 'wanting' - and knowing more about your 'wanting' - to doing.”*

*Although we do not ask people how many times they used WOOP, we do ask participants in the group discussion if their WOOP was successful and why, which does raise the issue of how much they are carrying it out (see S3d, page 5):*

*“Now, think about your wish. In your opinion, did you attain it? If so, how? If not, why?”*

*In these discussions, participants are mentioning if they are using WOOP although we do not ask how often specifically. Unfortunately, we have now started data collection and cannot add measures without deviating from our planned approach. Instead, we note the issue of the intervention ‘dose’ as a limitation:*

*“The dose of the intervention in one group session might be too weak and repeated practice or reminders over the study period could strengthen its effects.”*

**>>There is no recognition of the possibility of the participants being involved in other workplace interventions - this is a strong possibility especially as the workplaces being chosen are all those which are signed up for WHIs. It should be an exclusion criteria**

*Thank you for raising this important point. We agree it would be ideal to achieve full experimental control by excluding workplaces offering other interventions. However, all the workplaces in the study will be offering other interventions because they are participating in the local government health and wellbeing scheme. Our funding requires us to partner with the local government scheme to achieve policy impact.*

*The WOOP intervention sits alongside a menu of other initiatives in the scheme, such as walking clubs, anti-bullying policies, and free fruit and vegetables in kitchens. We unfortunately cannot exclude workplaces participating in the local government schemes because there would be no workplaces to participate in the study. While this is a weakness from an experimental design point of view, it does enhance the ecological validity of the study because it sits alongside ongoing initiatives. We now note this as a limitation:*

*“There could also be interactions between the intervention and other ongoing initiatives within the workplaces.”*

**>> In the first session the first action would be better and less prone to bias if the surveys were carried out prior to any discussion (as they are in the follow up session)**

*We carry out all surveys prior to any discussion (see Table 1 – a and b), which is now clarified in the text: “Note that all surveys are carried out prior to discussion in both sessions, meaning endpoint measures will be taken from the control group before they receive their intervention.”*

**>> I wonder if the demographic questions are using standardised questionnaires - asking yes/no to have you a disability seems that it may not yield very helpful information.**

*We updated the manuscript to reflect that if participants say they have a disability they are asked to expand. The demographic questions are drawn from the national census survey.*

**>>How confident are you in the participants remembering their goals (can you put in place a system to remind them?) and does it matter if they change their goal or achieve one and go onto another one? Can you capture that?**

*We are not sure if everyone will remember, and we have accounted for this in our questionnaire text:*

*“As a reminder, in the previous session, you may have been asked to tell us about something that you wished to do for your health and wellbeing. Do you remember being asked about this? Yes/No/Unsure. As a reminder: It was supposed to be something challenging, but feasible to achieve in around four weeks. It did not need to be work-related, but it should have been something that you thought that you could complete in around four weeks. What did you wish to do for your health and wellbeing? (if you do not remember, leave this blank).”*

*We agree it would be interesting to capture if participants change their goal or achieve one and go onto another one. However, the instructions ask participants to focus on their most important wish if they have several. Therefore, we focus on their most important wish. We do ask about their cognitive participation with WOOP (incorporate it into work life and life outside work). The focus on single wish is a limitation we now acknowledge: “Multiple doses for various wishes could be required to achieve results.”*

*The issue of reminders was also raised in our piloting and, as a result of these discussions, participants now take away a WOOP handout where they write down their WOOP (see S3f). Unfortunately, we did not have the resources to implement a more sophisticated reminder system with our budget. Another consideration was that not all employees were easily contactable for reminders - some of them do not have work email addresses (they might work as drivers or in factories). We decided not to ask them for their personal phone numbers or email addresses for privacy reasons. Thus, we decided to leave staff with the handouts and leave it up to them to use them.*

**>> In light of the difference between wish and goal, the title, and elsewhere in the text, would be better reworded to reflect this with something like 'wish derived goals'. It would be interesting to unpack this a bit more too.**

*Thank you for this comment. We agree it is important to further specify the link between wishes and goals to clarify our approach. In the existing literature on mental contrasting plus implementation intentions, wishes are potential goals, and wishes become goals once they are selected and people commit to them. Thus, upon reflection, we perceive this is a goal-based intervention because participants are asked to commit to their wishes, turn them into goals, and report on whether they were able to achieve them. Our title has been changed accordingly to goal-based rather than goal-setting and we have clarified our use of the language within the manuscript text.*

*To clarify our use of this language, the introduction, we now link wishes to goals by stating: “In applications of mental contrasting and implementation intentions, participants are asked to select from multiple potential goals (called wishes) and strengthen their commitment to them using these strategies.” We also now describe wishes and goals in the intervention section: “During the intervention activity, participants will be encouraged to use mental contrasting and implementation intention steps to reinforce the wish, turning it into a committed goal.”*

*We draw on text from Gollwitzer, P. & Oettingen, G. (2011):*

“The concept of implementation intentions grew out of a more comprehensive approach to goal pursuit: the mindset theory of action phases (Gollwitzer, 1990). The mindset model of action phases sees successful goal pursuit as solving a series of successive tasks: deliberating on wishes (potential goals) and choosing between them; planning and initiating goal-directed actions; bringing goal pursuit to a successful end; and evaluating its outcome. This task notion implies that people can activate cognitive procedures (mindsets) that facilitate task completion simply by getting heavily involved with the task at hand. Whereas deliberating between potential goals (i.e., wishes) activates cognitive procedures (i.e., a deliberative mindset) that facilitate decision making, engaging in planning activates those procedures (i.e., an implemental mindset) that support the implementation of goals (p. 177).”

*Gollwitzer, P. & Oettingen, G. (2011). Planning promotes goal striving. In K. Vohs & R. Baumeister (Eds.), Handbook of self-regulation: research, theory, and applications (pp. 162-185). ISBN 978-1-606-23948-3*

**>> The limitations of participant selection is partly acknowledged but there is also purposeful selection and the bias effect of this should be explained**

*We have modified the text to explain the effect of purposeful selection on generalisability:*

*“A further limitation is that employees volunteered to take part, which may introduce selection bias. Although a mitigation step is planned by asking workplaces to invite staff across diverse occupations in workplaces, purposeful selection in this way means that the results may not generalise to those who are not selected.”*

**>> Please clarify what 'all organisation involved in the research will receive extra help implementing content about health and wellbeing at work' - is this as an incentive to participate? Will it impact on the study at all?**

*The extra help is the intervention itself. We do not specify what the intervention is prior to delivering it to avoid participants going through WOOP before the session with us. However, our ethics team wanted us to clarify that something extra will take place with the research.*

**Reviewer #4: Review of Registered Report Protocol PONE-D-22-11119 submitted to PLOS ONE entitled: “A cluster randomised wait-list controlled trial of a goal-setting behavior change intervention for employees in workplaces enrolled in workplace health and well-being initiatives”.**

**Thank you for your invitation to review this study protocol.

I acknowledge and appreciate the authors’ willingness to adapt their methods in response to the feedback from Reviewers 1 and 2. Overall, this protocol is clear and well written. A strength of this study is the large group of workplaces included in the sample, and the use of a placebo.

The authors do not describe how “mental contrasting” and “implementation intentions” differ from “goal setting” and “strategies” which are commonly used and widely evaluated health promotion concepts, including in workplace settings. For example, Malik, S. H., Blake, H., & Suggs, L. S. (2014). A systematic review of workplace health promotion interventions for increasing physical activity. British Journal of Health Psychology, 19(1), 149–180.** [**https://doi.org/10.1111/bjhp.12052**](https://doi.org/10.1111/bjhp.12052)**. How does this study differ from these previous studies? As Reviewer 2 stated, mental contrasting focuses on a “wish” as opposed to a goal, yet the term goal is still used throughout this protocol, including in the title. A wish and a goal are distinctly different constructs and therefore using them interchangeably creates confusion for the reader, and diminishes the novelty of the study.**

*Thank you for this important comment, which also aligned with Reviewer 3’s comment on wish-derived goals (see above). We have changed the title and manuscript text to better address the relationship between wish and goal. We now also provide further detail on how mental contrasting and implementation intentions differ from goal setting and strategies as health promotion concepts.*

*To recap, our title has been changed from goal-setting to goal-based because mental contrasting and implementation intentions are more than about setting goals. Instead, these strategies also strengthen goal commitment and action planning, which are additional to the process of goal setting.*

*Drawing on Gollwitzer & Oettingen (2011), we now clarify the relationship between wishes and goals with the following text (see also above): “In applications of mental contrasting and implementation intentions, participants are asked to select from multiple potential goals (called wishes) and strengthen their commitment to them using these strategies.”*

**I agree with reviewer one that I have concerns about the authors’ capacity to evaluate effects on health behaviour using the methods described. You state in the abstract that “… rigorous effectiveness evaluations of them are rare. In this research we propose evaluating the effectiveness of an established behaviour change intervention in a new workplace context.” It appears that the study will not evaluate clinical effectiveness or objective measures of behaviour change, but only the participants’ perception of effectiveness over a short timeframe, and therefore I find this language to be an overstatement as it gives the impression that this is a rigorous effectiveness evaluation. If inclusion of objective outcome measures, such as examples or observations of behaviour change, is not possible, I encourage the authors to be more circumspect in their language.**

*We agree that we implied our evaluation was a rigorous effectiveness evaluation which is inconsistent with our reliance on self-report measures. We have removed the word rigorous from the Abstract and now pre-empt the reader in the Introduction to expect self-reported measures. However, we have kept the language of effectiveness evaluation because we include a control group and ask participants if they changed their behaviour.*

*The text changes to the Introduction now reads: “Our outcomes are self-reported and the intervention period lasts only several weeks, however, if positive effects are found, a more rigorous trial could consider including objective measures of health and extending the timeframe.”*

**The “post” data collection is only four weeks after the “intervention” – is there any intention to track behaviour change over a longer period? Sustaining a behaviour change is more difficult than creating a short-term change over a four-week period. Is there evidence that a behaviour change over a four-week period is clinically significant for a person’s health outcomes without subsequent follow-up?**

*Although we would like to be able to track participants beyond four weeks, we do not have the resources for a longer follow-up period due to funding limitations. We agree that sustained behaviour change is a challenge to achieve and there would be questions about the clinical significance of any changes, especially given the heterogeneity of the goals participants can set for themselves. Our limitations section now expands on this point: “If positive effects are found for measures of self-report, future research could examine the effects on observational measures like absenteeism or productivity, if any changes are sustained over the longer term, or if changes have clinically significant implications for health and wellbeing.”*

**Thank you for including sex and gender as separate variables.

P14-15 - I will leave it to other reviewers more familiar with the Bayesian approach described to comment on these methods.

P 13 and 15 - Qualitative methods – there is a lack of detail about the qualitative analysis methods for Please include more details about your qualitative analysis methods such as how many coders, how will you ensure inter-rater reliability, will transcripts be deidentified prior to analysis, how will you address any potential bias of the coders and their interpretation of the data? For example, are the coders the same people conducting the workshops? Are they independent researchers? Please refer to a relevant qualitative research reporting standard e.g., O’Brien, Bridget C. PhD; Harris, Ilene B. PhD; Beckman, Thomas J. MD; Reed, Darcy A. MD, MPH; Cook, David A. MD, MHPE. Standards for Reporting Qualitative Research: A Synthesis of Recommendations. Academic Medicine: September 2014 - Volume 89 - Issue 9 - p 1245-1251 doi: 10.1097/ACM.0000000000000388**

*Thank you for the opportunity to expand in detail about the qualitative analysis. As we are taking a reflexive thematic approach to identify patterns of shared meaning, we will not be evaluating inter-rater reliability (which is aligned with thematic analysis approaches using cluster and call coding reliability or thematic analysis with codebooks – see references 63-65). We will follow COREQ reporting standards and now specify this in our porotocol. Our updated text now reads:*

*“Analysis of qualitative data*

*Qualitatively, focus groups will be audio recorded, transcribed, and coded, and reflexive thematic analysis will be used to conceptualise analytic themes about barriers, enablers, and mechanisms surrounding the use of WOOP [63–65].* *We also analyse fieldnotes from focus groups. To inform the development of themes, will draw on the behaviour change wheel[24] to deductively shape our understanding of the data on barriers and enablers and on normalisation process theory to shape themes from the data on mechanisms.[51] Our coding approach will involve members of the research team who both did and did not collect the data, mixing informed and independent approaches, taking a collaborative and reflexive approach to identify patterns of shared meaning across the dataset [65]. Our results will be reported using the consolidated criteria for reporting qualitative research [66].”*

**Fidelity – you have responded to Reviewer 2 that you will now ask the workshop facilitators whether they delivered the intervention or control session as indicated in the envelope. Will you also ask about any adaptations made and the reasons for these, so that these are documented? Although facilitators can have the best possible intentions, adaptations do happen and should be recorded.**

*Thank you for this good idea and we have now updated our reporting from to ask about any adaptations made and the reasons for these. For sessions already conducted, session facilitators have been asked to update their forms.*

**Overall, I feel that the methods described have strengths, such as the large sample size, clear procedures which increase the likelihood of fidelity, and the variety of workplaces. However, there are also significant limitations related to the short timeframe between intervention and follow-up, and the lack of objective measures of behaviour change or clinical outcomes.**

*Thank you for your suggestions to improve our study.*
